# An Analytical Toolbox for Fast and Straightforward Structural Characterisation of Commercially Available Tannins

**DOI:** 10.3390/molecules26092532

**Published:** 2021-04-26

**Authors:** Lili Zhen, Heiko Lange, Claudia Crestini

**Affiliations:** 1 Department of Chemical Science and Technologies, University of Rome ‘Tor Vergata’, Via della Ricerca Scientifica, 00133 Rome, Italy; lili.zhen@alumni.uniroma2.eu; 2 CSGI-Center for Colloid and Surface Science, Via della Lastruccia 3, 50019 Sesto Fiorentino, Italy; 3 Department of Earth and Environmental Sciences, University of Milano-Bicocca, Piazza della Scienza 1, 20126 Milan, Italy; 4 Department of Molecular Sciences and Nanosystems, University of Venice ‘Ca’Foscari’, Via Torino 155, 30170 Venice Mestre, Italy

**Keywords:** condensed tannins, hydrolysable tannins, ^31^P NMR, HSQC, MALDI-ToF

## Abstract

Both condensed and hydrolysable tannins represent versatile natural polyphenolic structures exhibiting a broad range of activities that could be exploited in various fields including nutraceutics, cosmesis, consumer care, household and pharmaceutical applications. Various tannins are commercially available nowadays for use in such application fields. We have analysed a representative selection of commercially available condensed and hydrolysable tannins for structural features and purity. Using a combination of quantitative ^31^P NMR spectroscopy, HSQC measurements, MALDI-ToF analyses, gel permeation chromatography and wet chemical analysis, detailed structural characterisations and descriptions were possible, allowing for verification and falsification of claimed structural features.

## 1. Introduction

Giovannetti investigated the interactions between iron solutions and substances called ‘astringents’ in the middle of the 17th century, marking as such what is today seen as the onset of tannin research. In 1772, researchers identified the presence of an acid in these compounds. This acid was subsequently isolated by Scheele and shown to be gallic acid. At the end of the 18th and the beginning of the 19th century, the tannins were officially recognised as a discrete group of molecules that differ with respect to the gallic acid content [1,2]. They generally appear in various forms, ranging from white to off-white amorphous powders to shiny, almost colorless pasty substances that exhibit characteristic smells and astringent tastes. Tannins can be found in most of the higher plants around the globe but also in algal biomass [2], produced in almost all parts of the plant; i.e., seeds, roots, bark, wood and leaves; due to their fundamental role in defending against insects, infections, fungi, bacteria or simply predators [3]. One defense mechanism relies on the capability of tannins to irreversibly complex proteins [4]. This feature is also the cause for the massive use of these substances in Asian medicine [4,5,6]. They are also thought to be one of the effective components contributing to the fact that the risk of suffering from cardiovascular diseases and certain forms of cancer can be reduced by choosing diets rich in fruits and vegetables [7,8].

The rather wide range of functions comes together with a wide variety of tannin structures, commonly divided in tannins such as gallotannins and ellagitannins, that are soluble and cleavable into their components in hot water or in the presence of a tannin-specific enzyme, e.g., tannase, and that were thus termed ‘hydrolysable’ tannins, and non-hydrolysable tannins or flavones, a tannin species without carbohydrate moieties, termed proanthocyanidins, polyflavonoid tannins, catechol-type tannins, and pyrocatecholic-type tannins that are stable under these conditions and thus called ‘non-hydrolysable’ or ‘condensed’ tannins [1,2,9]. Aiming at the use of tannins in material sciences [10,11,12,13,14] or biomedical applications [7,15,16,17,18,19,20], the rather precise knowledge of the structure of a given tannin is a key factor. Depending on the application, additional knowledge regarding dominant impurities might be necessary. The archival literature lists an impressive number of research efforts in which such structural characterisation of tannins has been achieved using various analysis techniques and/or combinations thereof [21,22,23,24,25,26,27]. Standard methods for the elucidation of tannin structures include NMR analyses, analyses by MALDI-ToF and classical wet-chemical analyses. Several dedicated articles review achievements and point out the challenges connected to this topic [21,22].

**Figure 1:**
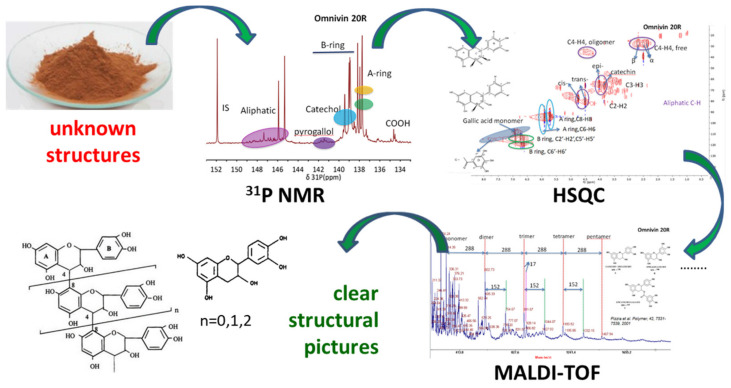
Analytical toolbox and workflow for elucidating structural features of commercially available tannins.

The design of novel products and processes aimed at improving the exploitation of the huge potential of tannins requires their use both in their natural form as well as in the form of chemically altered derivatives. It is therefore necessary, in order to develop targeted selective chemical derivatization protocols, to analyse the tannin structures, as such verifying also the information available for the commercialised samples. To this end, commercially available tannins from different origins have been structurally characterized using the toolbox shown in Figure 1: structural characterization was achieved by {^1^H-^13^C}-HSQC measurements, quantitative ^31^P NMR and ^1^H NMR spectroscopy, MALDI-ToF analysis and gel permeation chromatography (GPC).

Wet-chemical analysis such as the Scalbert test for the elucidation of cyanidin contents and the Folin–Ciocalteau assay for the determination of the total phenol content concluded the fingerprinting. On the basis of the results of the various analyses, it was possible to unequivocally assign the tannin structures. This is especially relevant for two reasons: (i) commercial tannins might be mixtures or even different from what is declared, and (ii) a detailed knowledge of structural features is key for an eventual chemical derivatisation of tannins.

## 2. Results and Discussion

As commercially available materials, eleven representative tannins from two different suppliers were selected (Table 1): condensed tannins Omnivin WG, Omnivin R, Omnivin 10R, Omnivin 20R, MIMOSA ATO ME, and QUEBARACHO ATO, as well as hydrolysable tannins Tanal 01, Tanal 02, Tanal 04, VEGETAN CN POLVERE and TARA POLV TIPO A. The tannin extracts were studied without any purification; i.e., as commercial-grade material.

The analysis techniques summarised as the toolbox shown in Figure 1 were applied to all tannins, and detailed data for each type of analysis performed are given in tables in the form of the Supplementary Materials. In the following paragraphs, only the structural outcomes of the combinatorial structural analyses are discussed, together with comments regarding structural peculiarities and eventually identified impurities.

### 2.1. Characterisation of Commercially Available Condensed Tannins

**Figure 2:**
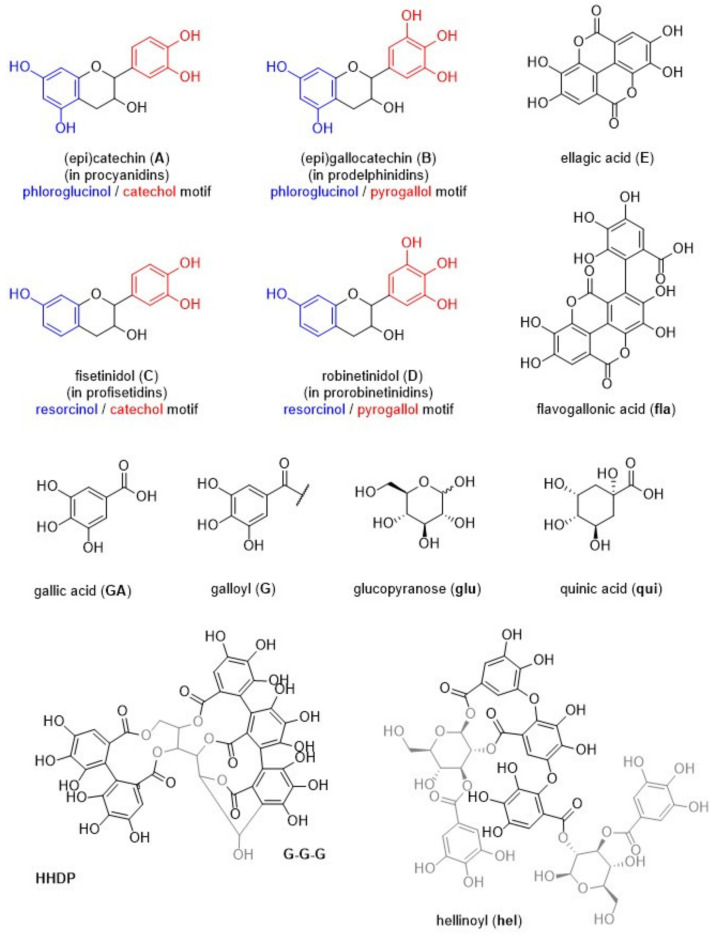
Structural elements used for discussing the various analysis data. Structures in grey are not name-giving.

NMR is a powerful tool for the analysis of natural polyphenols. The main issue in the analysis of tannins is represented by their high heterogeneity and variability. The ^1^H NMR signals are therefore not very diagnostic. On the contrary, ^31^P NMR of suitably in situ labelled samples allows direct straightforward identification and quantification of all the different phenolic groups present in the tannin sample. [27,28,29,30] This technique not only allows the study of tannins in complex matrices, but also provides at a glance the assignment of an unknown tannin to a hydrolysable, condensed or complex class. Furthermore, since it is possible to identify and quantify all the different phenolic groups present on the tannin backbone, it allows, in the case of proantocyanidins, assigning the substitution pattern in rings A and B. We started the structural analyses using different NMR spectroscopic techniques; the results of the quantitative ^31^P NMR analyses, delineated as described before, [27,28,29,30] are listed, together with the data for the other condensed tannins used in this study, in Table 2. The underlying spectra are shown in the Supplementary Materials in Figures S1A, S3A, S5A, S7A, S9A and S11A. Figure 2 shows structural elements identified and used for analysis and throughout the various structural discussions. Molecular mass analyses with the aim of delineating the dominating degree of polymerisation were possible in the form of gel permeation chromatography on non-acetylated samples in DMSO, and the results are listed in Table 2 as well. Although eventually of less structural potential, traditional wet-chemical analyses were performed on the various tannins in the form of Scalbert [31] and Folin–Ciocalteau [32] tests, and these results are also given in Table 2. Figures S1B–S6B in the Supplementary Materials show the HSQC spectra; Table S1 in the Supplementary Materials lists the assigned cross-peaks from the HSQC analyses of the various tannins on the basis of various literature reports [23,24,30,33,34]. A more detailed analysis of the nature and/or composition of monomeric tannins and tannin oligomers was achieved by MALDI-ToF analyses [24,35]; a full list of identified masses is given for each condensed tannin in Table S2 in the Supplementary Materials.

The various single data especially of the NMR analyses, flanked by the MALDI-ToF analyses, were used to cross-verify and deduce more intrinsic information of the sample, such as the ratio of the units found in terms of composition and the sample purity with respect to what could be theoretically expected. Table 3 summarises this information for the analysed condensed tannins.

#### 2.1.1. Characterization of Monomeric and Low Oligomeric Condensed Tannins

**Figure 3:**
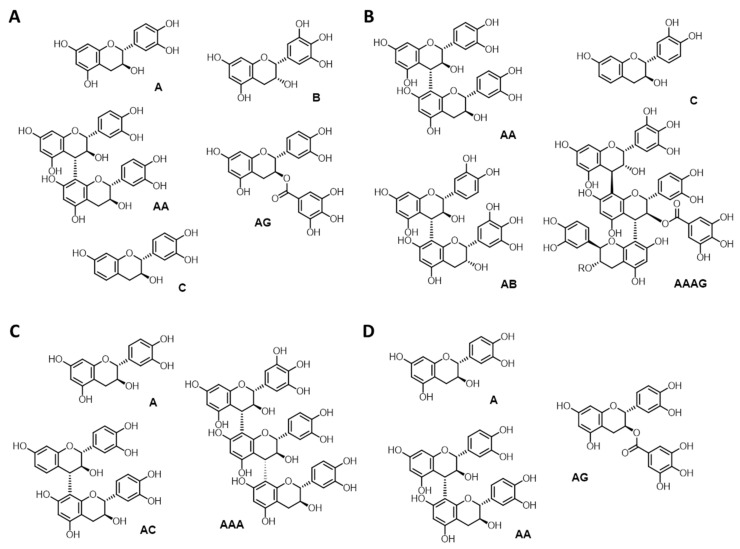
Representative examples of structural features identified on the basis of NMR and MALDI-ToF data in (**A**) Omnivin WG (***Vv***); (**B**) Omnivin R (***Vv*-R**); (**C**) Omnivin 10R (***Vv*-10**); and (**D**) Omnivin 20R (***Vv*-20**).

Omnivin WG (***Vv***) is sold as an average-grade wine tannin. According to the available accompanying documentation, this tannin should exhibit a larger polydispersity with respect to the other members of the ‘Omnivin’ family of tannins commercialised by the producer. By and large, structural analysis of ***Vv*** confirmed this. The sample is found to be present mainly in the form of procyanidins (62%) and profisetidins (34%). The ratio between phloroglucinol and resorcinol motifs as deduced from ^31^P NMR analysis seems to be reflected in the semiquantitative HSQC data. HSQC suggests that both catechin and epicatechin stereochemistries are present. On the basis of the HSQC analysis, an indicative cross-peak for a C4-H of a substituted C4 delineated low oligomeric character is confirmed by comparative GPC analysis (Table 2), with an estimated degree of polymerisation of 1–2, determined by the combination of GPC and MALDI-ToF data. Gallocatechin monomers are present only in minor quantities (Table 2). MALDI-ToF data suggest further that some molecules are esterified with gallic acid, which is also found to be present as impurity in free form in the HSQC spectrum, ^31^P NMR analyses (Table 2) in form of free acidic OH-groups and MALDI-ToF spectra. The overall sample purity, determined as described before comparing the theoretically expectable flavan-3-ol content in 1 g of substance to the ^31^P NMR-derived amount, was found to be 78%. Both ^31^P NMR and HSQC spectra correspondingly reveal signals that can be connected to carbohydrate impurities. Figure 3a shows the main structural representation of ***Vv*** based on these structural insights, trying to combine the quantitative ^31^P NMR data with the non-quantitative MALDI-ToF data.

Omnivin R; i.e., ***Vv*-R**; shows essentially identical structural features as ***Vv***, as should be expected. A slight increase in fisetinidol over (epi)catechin elements is, however, observed (Table 3), alongside a shift towards higher molecular weights by GPC and MALDI-ToF, which indicates a polymerisation degree up to 3. The sample purity in terms of flavan-3-ol units per gram was found to be significantly reduced to only 39%; correspondingly, ^31^P NMR and HSQC spectra reveal considerable amounts of carbohydrate impurities. Gallic acid is found once more as impurity. Structural details are given in Figure 3.

Omnivin 10R and Omnivin 20R; i.e., ***Vv*-10** and ***Vv***-**20**, respectively; are sold as wine tannins of higher refinement grades compared to ***Vv*** and ***Vv-R***, with ***Vv*-20** being the highest refined product with respect to polydispersity, structural purity and non-tannin impurities. Accumulative data analysis suggests that the polydispersity for ***Vv*-10** and ***Vv*-20** reduces significantly (Table 2), resulting in ***Vv*-20** being by and large monomeric (epi)catechin: only trace amounts of dimeric species and (epi)catechin gallates are present according to HSQC; i.e., the indicative cross-peak for a C4-H of a substituted C4 is missing; MALDI-ToF analysis confirms this in terms of peak intensities found for dimeric species. Traces of (epi)gallocatechin are found in case of ***Vv*-10**, but practically not in ***Vv*-20**. In terms of monomer composition, ***Vv*-10** and ***Vv*-20** are mainly catechins, containing only 26% and 21% fisetinidol, respectively. Sample purity is found to significantly higher in ***Vv*-20** with respect to ***Vv*-10**; i.e., 88 vs. 46%, respectively. Sample purity for ***Vv*-10** is thus only in the range of the less purified samples. While this is less obvious in the acquired HSQC spectrum, the strong signal intensities observed in the aliphatic region of the ^31^P NMR spectrum of ***Vv*-10** do support this finding. Figure 3c,d illustrate the structural results obtained for ***Vv*-10** and ***Vv*-20**,

#### 2.1.2. Characterisation of Higher Oligomeric Condensed Tannins

**Figure 4:**
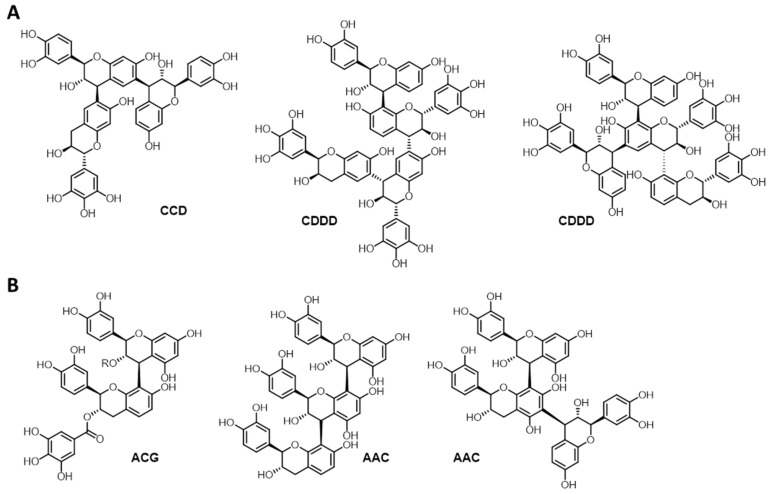
Structural representations delineated on the basis of NMR and MALDI-ToF data of (**A**) MIMOSA ATO ME (***Am***) and (**B**) QUEBRACHO ATO (***Sb***).

MIMOSA ATO ME (***Am***) is sold as a higher-grade oligomeric condensed tannin. Structural analysis of the batch used in this study confirms this, identifying ***Am*** by HSQC analysis and MALDI-ToF as an oligomeric tannin composed of approximately one third prorobinetidins and two thirds profisetidins. HSQC analysis further suggests that the *trans*-isomers prevail the *cis*-isomers for the various building blocks. Data also suggest that oligomerisation occurs more preferentially via C6 of the A-ring rather than via C8, given that the C8-H signal appears more intense in direct comparison. The cross-peak for C4-H substituted is stronger than the one for C4-H unsubstituted, underlining the oligimeric nature. Both ^31^P NMR and MALDI-ToF suggest that approximately 4% ***Am*** is esterified in position 3 of the C-ring with gallic acid. It is noteworthy that free gallic acid is only barely detectable in both MALDI-ToF ^31^P NMR and HSQC analysis; the latter suggests, however, together with the ^31^P NMR data, that ***Am*** contains significant amounts of carbohydrate impurities. The estimated sample purity amounts accordingly to approximately 51%. The structural findings are summarised in Figure 4a.

QUEBRACHO ATO (***Sb***) is sold as oligomeric condensed tannin. Data analysis suggests that the structure is composed of a rather unbiased mixture of profisetidin- and procyanidin-typical monomers; i.e., catechol and fisetinidol in a ratio of approximately 3/1; with some of the aliphatic OH-groups presumably esterified with gallic acid. NMR data indicate further the presence of very small amounts of epigallocatechin motifs. These would be, according to NMR, the only *cis*-configured motifs; all others seem to present exclusively as *trans* isomers. Interestingly, the de facto-absence of the C8-H proton signal in the HSQC suggests that monomers are practically exclusively connected in C4->C8 mode. MALDI-ToF and GPC analyses suggest oligomeric structures of mainly trimeric character, with tetramers being the largest reliably detectable units. Free gallic acid has been detected as impurity by MALDI-ToF, ^31^P NMR and HSQC. Additionally, in the case of ***Sb*** carbohydrate, impurities are delineable by ^31^P NMR and HSQC analyses. A structural representation of ***Sb***, found to have a purity of 50%, is given in Figure 4b.

The Scalbert test shows interesting results: the lowest cyanidin content was found for the highest refined sample ***Vv*-20**, which was proven to be of higher purity by HSQC, ^31^P NMR and MALDI-ToF analyses. The highest values were found instead for the non-refined *Vitis vinifera* sample ***Vv*** and the quebracho tannin (***Sb***) (Table 2). Since the test is known to be relatively easy to be compromised by impurities, the overall results indicate that the mere determination of the cyanidin content is eventually inadequate for determining the quality in terms of the structural purity of a sample, or eventually even the successful outcome of a refinement of a sample. The solubility of samples can represent a major source of error in these measurements; however, the test conditions chosen for this study were capable of solubilising all samples adequately.

**Figure 5:**
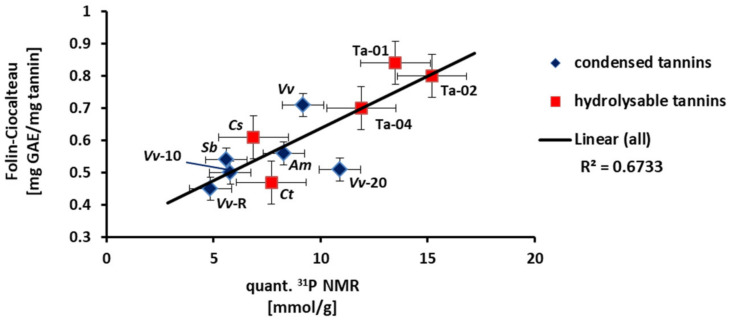
Correlation between the results obtained for the total phenol content by means of quantitative ^31^P NMR and the Folin–Ciocalteau method.

Calling for the overall phenolic OH-group content, the Folin–Ciocalteau test correlates roughly with the total phenolic OH-group content as determined by quantitative ^31^P NMR (Table 2, Figure 5). Since solubility issues can be excluded as major error source-chosen concentrations allowed for complete dissolution, possible sources for the error might lie in the impurities that were detected in the various samples in various amounts.

The results of the detailed structural analysis of the different commercialised condensed tannins correspond by and large to what could be expected based on knowledge in the archival literature. An interesting question in case of oligomeric tannins is the connectivity between the monomeric units. This question arises on the one hand in the form of the possibility of a 4→6 or a 4→8 linkage. This can eventually be estimated by HSQC, and more generally NMR-based analysis, as discussed above in case of ***Am*** and ***Sb***. In case indications for both linkages are found, it is more difficult to judge whether the two linkages connect the very same A ring of a central flava-3-ol to two other terminal units, or whether a chain is formed in which A rings carry only one linkage. Such more refined structural characterisations are principally possible using HSQC; however, given that the tannins come in mixtures of monomers and small oligomers, it is practically unfortunately rather impossible. The structural representation of the various tannin structures therefore lists various possibilities for some of the motifs identified in the MALDI-ToF analyses.

The difference might be reflected in the surface characteristics of the different molecules, and thus also in their biological activities beyond pure structural features such as the molecular weight or total number of OH-groups. Apart from reactivities of A-ring phenolic OH-groups certainly changing as a function of the substitution pattern, the question is additionally complicated by the possibility of a branching as soon as more than three monomeric units are linked. Such branching is reported especially in the case of 5-deoxyflavan-3-ol units being present, as in the case of the investigated ***Am*** and ***Sb*** [1,25,30,35,36]. Linear or branched structures will have different characteristics. One of the differences should be found in terms of the hydrodynamic volume, and as such, branching might explain why the molecular weight features as determined by GPC, which reflects also hydrodynamic volume characteristics of the analytes, does not show more significant differences compared to the monomeric and dimeric Omnivin tannins.

### 2.2. Characterisation of Commercially Available Hydrolysable Tannins

In case of commercially available hydrolysable tannins, it is worthwhile to understand the nature of the tannin, especially also with respect to the core sugar moiety. Tannic acid (**TA**) is probably the most commonly available hydrolysable gallotannin; however, archival literature indicates that **TA** exists in the form of various derivatives of 1,2,3,4,6-penta-*O*-{3,4-dihydroxy-5-[(3,4,5-trihydroxybenzoyl)oxy]-benzoyl}-d-glucopyranose that could be seen as the ‘default’ **TA**. Several other hydrolysable tannins exist, of course, varying in terms of the core carbohydrate, the repeat number of esterified galloyl units, the form and degree of additional oxidative linkages between the aromatic moieties and eventual oligomeric gallic acid-stemming impurities.

As for the condensed tannins, the combination of analysis techniques listed in Figure 1, with the exception of GPC, was used for delineating the structural peculiarities of five commercialised hydrolysable tannins. Table 4 lists the results obtained for the ^31^P NMR-based quantitation of hydroxyl groups contents and results of wet-chemical analysis according to the Scalbert and Folin–Ciocalteau tests. In the Supplementary Materials, Table S3 gives an overview of structural motifs identified by HSQC measurements and Table S4 an overview of the results by MALDI-ToF analysis.

Tanal 01 (**Ta-01**) is commercialised as a typical tannic acid, and so are Tanal 02 and Tanal 04, with the differences lying in molecular size and phenolic content. Such differences are principally compatible with a ‘typical’ tannic acid structure in the sense that such a ‘typical’ tannic acid does not exist, but that the number of galloyl units attached to the centre carbohydrate may vary. It was thus initially expected to confirm within the structural analysis the presence of glucose as common central sugar in all three Tanal samples under study.

**Figure 6:**
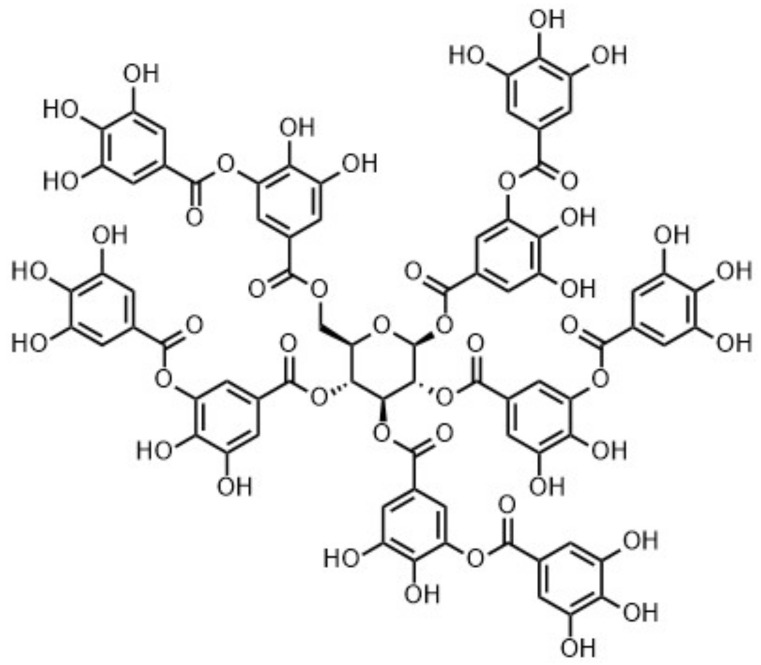
Structural representation of Tanal 01 (**Ta-01**) and Tanal 02 (**Ta-02**). Structural ‘impurities’ such as hellinoyl and flavogallonic acid are not indicated.

Most interestingly, however, only Tanal 01 (**Ta-01**) and Tanal 02 (**Ta-02**) turned out to be tannic acids; i.e., displaying a glucopyranose core esterified with 10 galloyl units (Figure 6). Tanal 02 was found to be essentially identical in structure to Tanal 01 (Table S3), with little differences in terms of the detectable presence of flavogallonic acid. Both tannins were found to contain hellinoyl units as a ‘structural impurity’ (Tables S3 and S4).

**Figure 7:**
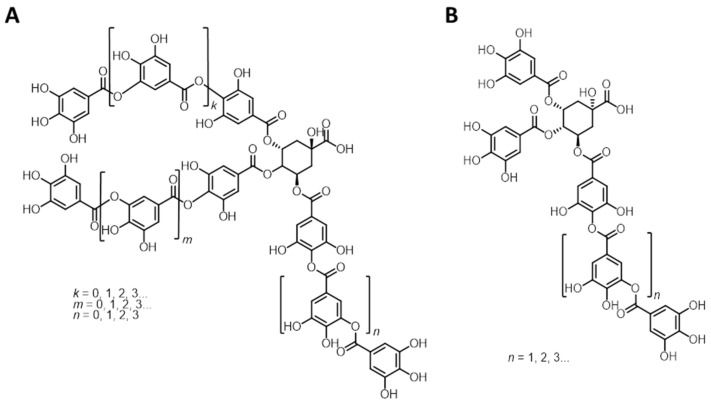
Structural representation delineated on the basis of NMR and MALDI-ToF data of (**A**) Tanal 04 (**Ta-04**) and (**B**) TARA POLV TIPO A (***Ct***).

Tanal 04, **Ta-04**, on the other hand, was found to represent a galloquinic acid. The NMR data are clearly distinctive with respect to **Ta-01** and **Ta-02**. MALDI-ToF analysis, however, was suitable to confirm the quinic acid structure: the analysis suggests a quinic acid core esterified with three to 12 galloyl units in total. Delineable structural features are displayed in Figure 7a.

In terms of molecular weights, the two tannic acids and **Ta-04** show similar data, underlining that it is not possible to distinguish between these hydrolysable tannins by rather blunt methods such as GPC. All three Tanal samples were found to contain free gallic acid, ellagic acid and flavogallonic acid as impurities detectable by NMR techniques; i.e., at levels above approximately 5%; MALDI-ToF confirms these findings (Table S4).

Another galloquinic acid was found in the form of TARA POLV TIPO A (***Ct***), a commercialised tara tannin. HSQC analysis clearly indicates the presence of a quinic acid as the core carbohydrate (Table S3). As indicated in Figure 7b, this tara tannin was found to comprise up to nine galloyl units linked to the quinic acid core in MALDI-ToF analyses (Table S4). ^31^P NMR analysis revealed higher quantities of carbohydrate impurities, easily identifiable by the high intensity of the signal corresponding to phosphitylated aliphatic OH-groups.

**Figure 8:**
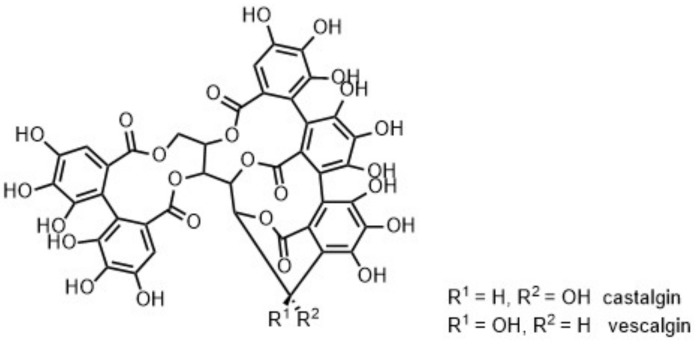
Structural representation delineated on the basis of NMR and MALDI-ToF data of VEGETAN CN POLVERE (***Cs***).

VEGETAN CN POLVERE (***Cs***) is a commercialised chestnut tannin. The batch analysed in this work turned out to be a mixture of castalgin and vescalgin, as indicated in Figure 1 and Figure 8. Both characteristic cross peaks in the HSQC analysis as well as MALDI-ToF analysis indicate these structures (Tables S3 and S4); quantitative ^31^P NMR data confirm this interpretation as well. The latter analysis additionally indicates also in this case noteworthy amounts of carbohydrate impurities.

Analysis of the molecular weight features of the bulk material in the form of GPC measurements in DMSO failed under the chosen conditions in the case of the hydrolysable tannins. The number average molecular weights of the two tannic acids under study; i.e., **Ta-01** and **Ta-02**; that were determined using the standard method, yielded numbers that need to be considered an underestimation of the real molecular weight estimable on the basis of elusive NMR and MALDI-ToF results. A reason for this could be the large number of aromatic units present in the structure. Earlier studies on phenolic structures suggested that high amounts of aromatic structures could lead to underestimation of Mn [37].

Wet-chemical analysis in the form of Scalbert and Folin–Ciocalteau assays indicated, as could be expected, insignificant cyanidine contents for tannic acids **Ta-01** and **Ta-02**; for **Ta-04**, the first quinic acid, cyaniding equivalents are detectable, above noise level, with 2 mg per gram material. Still higher amounts of approximately 10 mg per gram material are found for the other galloquinic acid ***Ct*** and the castalgin/vescalgin mixture. In light of the other analyses, the values can be seen as another example of the fact that impurities can impact the outcome of the Scalbert test.

The Folin tests follow the trend from the ^31^P NMR analyses and indicate the highest total phenol contents for the tannic acids (Table 4); the correlation between the results obtained by quantitative ^31^P NMR on phosphitylated samples and the values obtained for the Folin–Ciocalteau test was found to be higher than in the case of the condensed tannins (Figure 5). Galloquinic acid **Ta-04** shows a slightly reduced phenol content, but much more than galloquinic acid isolated as tara tannin, a fact that reflects the presence of higher amounts of carbohydrate impurities as revealed by ^31^P NMR. The VEGETAN sample ***Cs*** contains the lowest phenolic content in ^31^P NMR; the Folin test, however, suggests a content similar to that of **Ta-04**. Most likely, impurities are responsible for this outcome.

## 3. Materials and Methods

### 3.1. General Information

Reagents and solvents were purchased and used without further purification, if not stated otherwise, from Sigma Aldrich and Carlo Erba. Tannins were purchased from various vendors, as listed in Table 1, and used without further purification if not stated otherwise.

### 3.2. Nuclear Magnetic Resonance (NMR) Measurements

*^31^P NMR measurements:* The previously described procedure was followed [27,28,29,30]. In brief, approximately 15 mg of tannin were accurately weighed and added to 450 µL of a mixture of pyridine/CDCl_3_ (1.6:1). One hundred microliters of the standard solution, prepared using *N*-hydroxy-5-norbornene-2,3-dicarboxylic acid imide (***e*-HNDI**) at a concentration of 0.1 m in the above-mentioned solvent mixture mixed with 50 mg/mL of chromium(III) acetylacetonate as spin-relaxing agent were added, followed by 50 µL of 2-chloro-4,4,5,5-tetramethyl-1,3,2-dioxaphospholane (**Cl-TMPD**). After 1 h stirring at room temperature, the functionalized mixture was quantitatively transferred to a standard NMR tube for analysis. ^31^P NMR spectra were recorded on a Bruker 400 MHz spectrometer at 20 °C using an inverse gated decoupling sequence with a delay of 10 s between successive pulses. Chemical shifts were expressed in parts per million from 85% H_3_PO_4_ as an external reference. All chemical shifts reported are relative to the peak for the reaction product of water with **Cl-TMDP** 132.2 ppm in the used conditions. NMR data were processed with MestreNova (Version 8.1.1, Mestrelab Research).

*^1^H-^13^C HSQC measurements:* Samples of around 50 mg were dissolved in 600 μL of DMSO-*d*_6_ (providing NMR sample solutions with concentrations of around 83 mg/mL); chromium(III) acetylacetonate was added as a spin-relaxing agent at a final concentration of ca. 1.5–1.75 mg/mL. HSQC spectra were recorded at 27 °C on a Bruker 700 MHz instrument equipped with TopSpin 2.1 software. Spectra were referenced to the residual signals of DMSO-*d*6 (2.49 ppm for ^1^H and 39.5 ppm for ^13^C spectra). ^1^H-^13^C HSQC spectra were obtained using 32 scans obtained using the standard Bruker pulse program (hsqcegtpsisp2) with the following parameters for acquisition: TD = 2048 (F2), 512 (F1); SW = 13.0327 ppm (F2), 160 ppm (F1); O1 = 4200.54 Hz; O2 = 14083.02 Hz; D1 = 2 s; CNST2 = 145; acquisition time F2 channel = 112.34 ms; F1 channel = 8.7102 ms. Processing: SI = 1024 (F2, F1), WDW = QSINE, LB = 1.00 Hz(F2), 0.30 Hz (F1); PH_mod = pk; baseline correction ABSG = 5 (F2, F1), BCFW = 1.00 ppm, BC_mod = quad (F2), no (F1); linear prediction = no (F2), LPfr (F1). The integration ranges as previously reported were applied. The NMR data were processed with MestreNova (Version 8.1.1, Mestrelab Research).

### 3.3. Matrix-Assisted Laser Desorption/Ionization–Time-of-Flight (MALDI-ToF) Mass Spectrometry

MALDI-ToF analyses were performed using a Voyager-DE™ PRO Biospectrometry™ Workstation operated using the Voyager operating software (version X). Samples were dissolved in water/acetone (4 mg/mL, 50/50 volume), and the solutions were mixed with the 2,6-dihydroxy-benzoic acid (**2,6-DHB**) matrix solution (10 mg/mL in acetone). For non-ionic analytes, to enhance ion formation, sodium chloride (NaCl) was added to the 2,6-dihydroxy-benzoic acid (**2,6-DHB**) solution (10 mg/mL in distilled water). The sample and the matrix solutions were mixed as follows: three parts matrix solution, three parts sample solution, one part NaCl solution; approximately 2.5 µL of the resulting mix was placed on the MALDI sample holder. After drying overnight in the dark, the samples were analysed using settings specifically optimised for each sample type. Assignments of mass peaks were achieved using (combinations of) the molecular mass(es) of the structural units shown in Figure 2.

### 3.4. Gel Permeation Chromatography (GPC)

Approximately 3 mg of tannin sample were dissolved in 1 mL DMSO containing 0.1% lithium chloride. A Shimadzu instrument was used consisting of a controller unit (CBM-20A), a pumping unit (LC 20AT), a degasser (DGU-20A3), a column oven (CTO-20AC), a diode array detector (SPD-M20A) and a refractive index detector (RID-10A); the system was controlled by Shimadzu LabSolutions (Version 5.42 SP3). Three analytical GPC columns (each 7.5 × 30 mm) in series were used for analyses: Agilent PLgel 5 µm 10,000 Å, followed by Agilent PLgel 5 µm 1000 Å, followed by an Agilent PLgel 5 µm 500 Å. HPLC-grade DMSO (Chromasolv^®^, Sigma-Aldrich) was used as an eluent at 70 °C column temperature. The run time at 0.25 mL min^−1^ flow rate was 20 min. Molecular weights were calculated from a linear calibration constructed with poly(styrene sulfonic acid) polymers (4300−2.6 × 10^6^ g mol^−1^); analyses were run in duplicate.

### 3.5. Determination of Cyanidin Content (Scalbert Test)

The standard procedure as described elsewhere was adapted [31]: 5.0 mL of an acidic ferrous solution (77 mg FeSO_4_·7 H_2_O in 500 mL HCl/*n*-C_4_H_9_OH (2/3)) were added to 0.5 mL of the aqueous tannin solution (1 mg/mL). The mixtures were heated at 95 °C during 15 min. The absorbance was read at λ = 530 nm employing a Shimadzu UV-1800 spectrophotometer, operated via UV Probe, Version 2.42. The results are expressed in cyanidin equivalents (CyaE) as mg per g of dry tannin material (mg CyaE/g tannin material) (ε mol (cyanidin) = 34,700 L mol^−1^cm^−1^) using Equation (1) with A—absorbance measured, V—volume, D—dilution factor, MW—molecular weight of cyanidin, ε—molar extinction coefficient cyanidin, l—path length, m—mass dry bark.

(1) $$\frac{\mathrm{mg}\;\left(\mathrm{CyaE}\right)}{g\;\left(\mathrm{sample}\right)}=\frac{A\;V\;D\;\mathrm{MW}\;*\;1000\;}{\varepsilon \;l\;m}$$

### 3.6. Determination of Total Phenolic Content (Folin-Ciocalteau Test)

The total phenolic content of the extracts was determined by the Folin–Ciocalteu method [32]: 2.5 mL of Folin reagent (diluted 10 times) was added to 0.5 mL of a solution of tannin in distilled water (0.1 mg/mL) or 0.5 mL of an aqueous solution of re-dissolved accurately weighed fibrous matrix (0.3 cm^2^/mL). After 2 min, 2.0 mL of sodium carbonate (7.5% (m/m)) were added. The mixture was heated at 50 °C for 5 min. The absorbance was read at λ = 765 nm employing a Shimadzu UV-1800 spectrophotometer, operated via UV Probe, Version 2.42. Calibration was achieved following this approach using a solution of gallic acid in distilled water (0.5 mg/mL), diluted to eight calibration points covering the range between 0 and 0.01 mg/mL for which a linear calibration curve was obtained. The results are reported as mg of gallic acid equivalent (GAE) per mg of dry tannin (mg GAE/mg tannin).

## 4. Conclusions

By employing a combination of analysis techniques well established in connection with the structural characterisation of tannins [21,22], such as HSQC and MALDI-ToF analysis, and quantitative ^31^P NMR [27], it was possible to determine the dominating structures of eleven commercialised tannin samples. By and large, the structural elucidation is in accordance with the structural discussions found in the manifold archival literature regarding the investigated tannins. More importantly, however, the detailed analysis allowed for veryfying the nature of the tannin as proposed by the vendor. The analysis gave an insight into the quality of the various samples in terms of impurities. Carbohydrates and small molecular weight gallic acid-stemming compounds were identified as the main sources of impurity. ^31^P NMR allows the direct structural assignment of tannins not only as pure compounds but also when present in complex matrices highly polluted by impurities. Based on the structural information obtained, the tannins can be used for chemical derivatisation and biological testing, as will be reported in due course.

## Figures and Tables

**Table 1:** Commercially available tannins used for a detailed structural characterisation.

Samples (Species)	(Presumable) Tannin Type	Supplier	Code
Omnivin WG (*Vitis vinifera*)	condensed	OmniChem	***Vv***
Omnivin R (*Vitis vinifera*)	condensed	OmniChem	***Vv*-R**
Omnivin 10R (*Vitis vinifera*)	condensed	OmniChem	***Vv*-10**
Omnivin 20R (*Vitis vinifera*)	condensed	OmniChem	***Vv*-20**
MIMOSA ATO ME (*Acacia mearnsii)*	condensed	Figli di Guido Lapi	***Am***
QUEBARACHO ATO (*Schinopsis balansae*)	condensed	Figli di Guido Lapi	***Sb***
Tanal 01 (unknown)	hydrolysable (gallotannin)	OmniChem	**Ta-01**
Tanal 02 (unknown)	hydrolysable (gallotannin)	OmniChem	**Ta-02**
Tanal 04 (unknown)	hydrolysable (gallotannin)	OmniChem	**Ta-04**
TARA POLV TIPO A (*Caesalpina tinctoria*)	hydrolysable (gallotannin)	Figli di Guido Lapi	***Ct***
VEGETAN CN POLVERE (*Castanea sativa*)	hydrolysable (ellagitannin)	Figli di Guido Lapi	**Cs**

**Table 2:** Results of quantitative ^31^P NMR analyses of phosphitylated commercially available condensed tannins, number average molecular weights and polydispersities as well as cyanidin contents and total phenolic OH-group content. Errors vary depending on signal overlap (see Supplementary Materials); the average error is 0.5 mmol/g.

Cond. Tannin	OH-Groups Content [Mmol/g]								MN [Da] (PD)	SCAL-bert ^c^	Folin ^d^
	Aliphatic	A-ring		B-ring		Total Phenol ^b^	Gallate ^a^	Acidic			
		*Ortho*-Phenol	Free Phenol	Pyro-Gallol [a]	Catechol ^a^						
***Vv***	4.22	0.88	0.50	0.11	2.64	9.18	0.50	0.55	300 (7.1)	176	0.71
***Vv-R***	7.61	0.23	0.28	0.08	1.38	4.86	0.32	0.59	500 (6.6)	145	0.45
***Vv-10***	7.49	0.69	0.31	0.07	1.62	5.79	0.31	0.59	400 (7.0)	156	0.50
***Vv-20***	2.57	3.04	0.47	0.00	3.06	10.9	0.25	0.47	300 (3.2)	113	0.51
***Am***	5.97	0.00	0.61	1.54	1.85	8.28	0.27	0.12	400 (2.4)	135	0.56
***Sb***	3.40	0.84	0.35	0.12	1.78	5.60	0.23	0.30	500 (3.9)	189	0.54

^a^: Abundance of motif as a whole; i.e., pyrogallol with 3 OH-groups, catechol with 2 OH-groups. ^b^: Value over complete phenolic shift range (144.00 to 137.00 ppm). ^c^: Cyanidin content according to Scalbert’s test in [CyaE mg/g] [31]. ^d^: Total phenol content according to the Folin–Ciocalteau assay in [mg GAE/mg tannin] [32].

**Table 3:** Structural features of the A and B Rings, as well as flavan-3-ol content in commercially available condensed tannins as delineable by ^31^P NMR spectroscopy and GPC analyses. Structural purity has been determined as described before [30]; gallate ester content is estimated based on the same molecular weight.

Sample	A Ring Structure Ratio Phloroglucinol/ Resorcinol	B Ring Structure Ratio Pyrogallol/ Catechol	Deduced Tannin Structural Features (%) (Procyanidin, Profisetidin, Prorobinetinidin, Prodelphinidin and/or Corresponding Monomers)	DP	Sample Purity (% Flavanol Content)
***Vv***	1.76/1	0.04/1	62% (epi)catechin/procyanidin (**A**), 34% fisetinidol/profisetidin (**C**), 4% gallocatechin (**B**) motifs, traces of 3-*O*-gallates	1–2	78
***Vv*-R**	0.81/1	0.05/1	43% (epi)catechin/procyanidin (**A**), 52% fisetinidol/profisetidin (**C**), 5% gallocatechin (**B**) motifs, traces of 3-*O*-gallates	1–3	39
***Vv*-10**	2.23/1	0.04/1	70% catechin/procyanidin (**A**), 26% fisetinidol/profisetidin (**C**), 4% gallocatechin (**B**) motifs, traces of 3-*O*-gallates	1–3	46
***Vv*-20**	6.49/1	0.00/1	87% catechin/procyanidin (**A**), 13% fisetinidol/profisetidin (**C**), practically no gallocatechin (**B**) traces of 3-*O*-gallates	1–2	88
***Am***	0.01/1	0.54/1	65% fisetinidol/profisetidin (**C**), 35% robinetinidol/prorobinetinidin (**D**), approx. 4% 3-*O*-gallates	2–4	51
***Sb***	0.39/1	0.07/1	25% catechin/procyanidin (**A**), 69% fisetinidol/profisetidin (**C**), 6% epigallocatechin (**B**/robinetinidol (**D**) motifs, traces of 3-*O*-gallates	2–3	50

**Table 4:** Results of quantitative ^31^P NMR analyses of phosphitylated commercially available hydrolysable tannins, number average molecular weights and polydispersities as well as proanthocyanidin contents and total phenolic OH-group content.

Hydrolysable Tannins	OH-Groups Content [mmol/g]							Scalbert [c]	Folin [d]
	Aliphatic	Internal Gallate	Terminal Gallate	Catechol [a]	*Ortho*- Substituted Phenol	Total Phenol [b]	Acidic		
**Ta-01**	**0.59**	2.20	2.51	3.27	4.58	13.5	0.22	0.55	0.84
**Ta-02**	1.03	2.68	2.61	2.93	5.60	15.2	0.15	0.27	0.80
**Ta-04**	0.92	2.21	1.84	3.38	3.06	11.9	0.15	1.91	0.70
***Ct***	3.01	2.64	0.28	3.37	1.34	7.70	0.18	9.01	0.47
***Cs***	4.77	2.90	1.14	2.21	0.74	6.87	0.32	10.6	0.61

a: Abundance of motifs as a whole; i.e., pyrogallol with 3 OH-groups, catechol with 2 OH-groups. b: Value over complete phenolic shift range (144.00 to 137.00 ppm). c: Cyanidin content according to Scalbert’s test in [CyaE (mg/g] [31]. d: Total phenol content according to the Folin–Ciocalteau assay in [mg GAE/mg tannin] [32].

## Data Availability

All data is reported in the main article or in the Supplementary Materials.

## Supplementary Materials

Figure S1: NMR analyses of Omnivin WG (***Vv***): (A) ^31^P NMR; (B) ^1^H-^13^C HSQC, Figure S2: MALDI-ToF analysis of Omnivin WG (***Vv***). Figure S3: NMR analyses of Omnivin R (***Vv-R***): (A) ^31^P NMR; (B) ^1^H-^13^C HSQC. Figure S4: MALDI-ToF analysis of Omnivin R (***Vv-R***). Figure S5: NMR analyses of Omnivin 10R (***Vv*-10**): (A) ^31^P NMR; (B) ^1^H-^13^C HSQC. Figure S6: MALDI-ToF analysis of Omnivin 10R (***Vv*-10**). Figure S7: NMR analyses of Omnivin R20 (***Vv*-20**): (A) ^31^P NMR; (B) ^1^H-^13^C HSQC. Figure S8: MALDI-ToF analysis of Omnivin 20R (***Vv*-20**). Figure S9: NMR analyses of MIMOSA ATO ME (***Am***): (A) ^31^P NMR; (B) ^1^H-^13^C HSQC. Figure S10: MALDI-ToF analysis of MIMOSA ATO ME (***Am***). Figure S11: NMR analyses of QUEBRACHO ATO (***Sb***): (A) ^31^P NMR; (B) ^1^H-^13^C HSQC. Figure S12: MALDI-ToF analyses of QUEBRACHO ATO (***Sb***). Figure S13: NMR analyses of Tanal 01 (**Ta-01**): (A) ^31^P NMR; (B) ^1^H-^13^C HSQC. Figure S14: MALDI-ToF analyses of Tanal-01 (**Ta-01**). Figure S15: NMR analyses of Tanal 02 (**Ta-02**): (A) ^31^P NMR; (B) ^1^H-^13^C HSQC. Figure S16: MALDI-ToF analyses of Tanal-01 (**Ta-02**). Figure S17: NMR analyses of Tanal 04 (**Ta-04**): (A) ^31^P NMR; (B) ^1^H-^13^C HSQC. Figure S18: MALDI-ToF analyses of Tanal-01 (**Ta-04**). Figure S19: NMR analyses of TARA POLV TIPO A (***Ct***): (A) ^31^P NMR; (B) ^1^H-^13^C HSQC. Figure S20: MALDI-ToF analyses of TARA POLV TIPO A (***Ct***). Figure S21: NMR analyses of VEGETAN CN POLVERE (***Cs***): (A) ^31^P NMR; (B) ^1^H-^13^C HSQC. Figure S22: MALDI-ToF analyses of VEGETAN CN POLVERE (***Cs***). Table S1: Results of qualitative ^1^H-^13^C HSQC analyses of commercialised condensed tannins according to literature reports. Table S2: MALDI-ToF analysis of commercialised condensed tannins. Table S3: Results of qualitative ^1^H-^13^C HSQC analyses of commercialised hydrolysable tannins according to literature reports. Table S4: MALDI-ToF analysis of commercialised hydrolysable tannins.
