# Supplementary material for: An Analytical Toolbox for Fast and Straightforward Structural Characterisation of Commercially Available Tannins
Source: Molecules. 2021 Apr 26;26(9):2532. doi: 10.3390/molecules26092532 (PMC8123674; doi:10.3390/molecules26092532)
Supplement: Supplementary file 1 [file molecules-26-02532-s001.zip › molecules-1170828-supplementary.pdf]

Supplementary Material

An Analytical Toolbox for Fast and Straightforward Structural Characterisation of Commercially Available Tannins

Lili Zhen [1,2], Heiko Lange [2,3,†,*], Claudia Crestini [2,4,†,*]

1 Department of Chemical Science and Technologies, University of Rome 'Tor Vergata', Via della Ricerca Scientifica, 00133 Rome, Italy

2 CSGI - Center for Colloid and Surface Science, Via della Lastruccia 3, 50019 Sesto Fiorentino, Italy

3 Department of Earth and Environmental Sciences, University of Milan-Bicocca, Piazza della Scienza 1, 20126 Milan, Italy

4 Department of Molecular Sciences and Nanosystems, University of Venice 'Ca'Foscari', Via Torino 155, 30170 Venice Mestre, Italy

† Affiliated with 1 *via* NAST – Nanoscience & Nanotechnology & Innovative Instrumentation Center.

* Correspondence: heiko.lange@unimib.it; claudia.crestini@unive.it

Number of pages: 29

Number of figures: 22

Number of tables: 4

Analysis data for commercialised condensed tannins

(A)

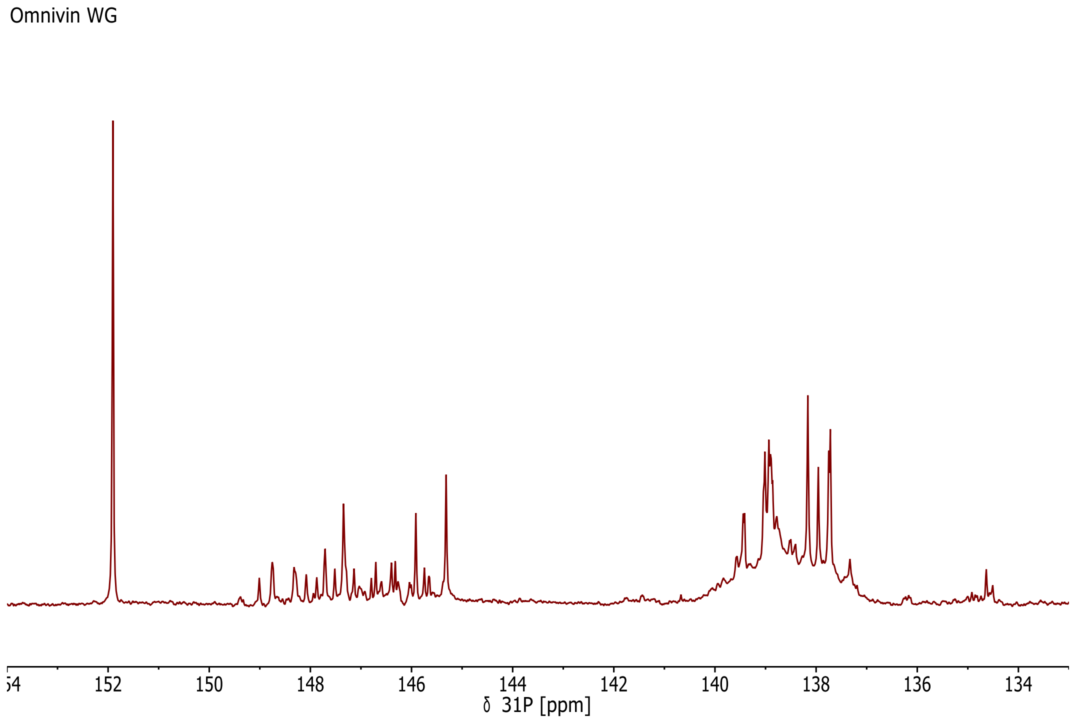

(B)

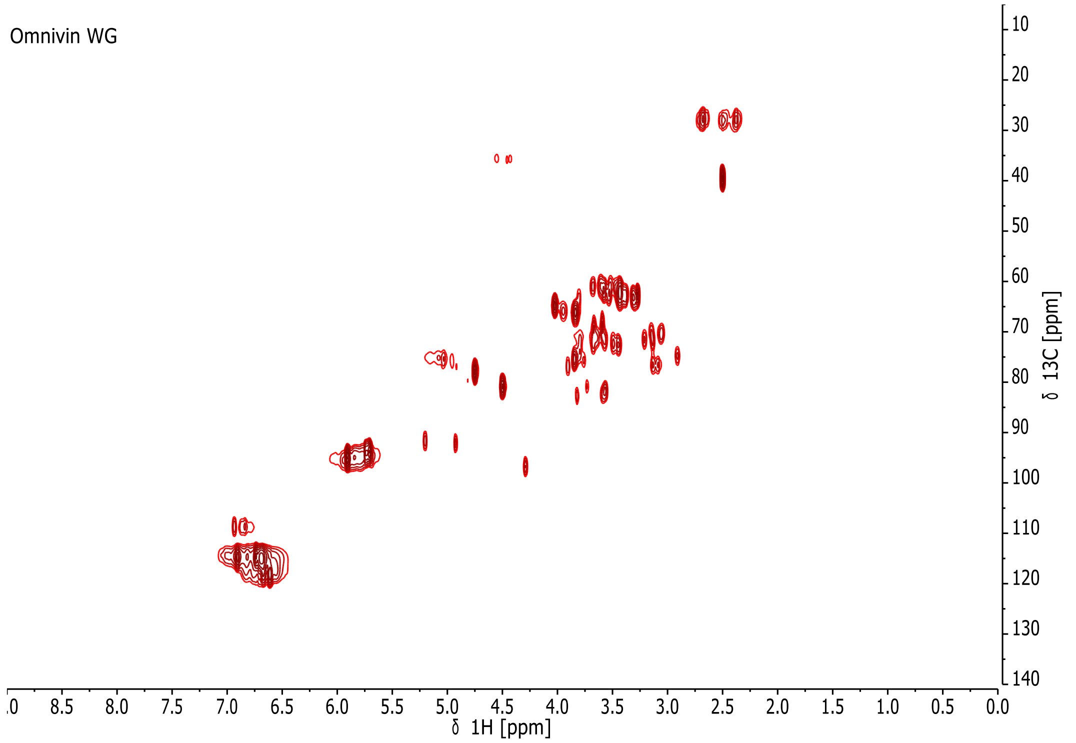

Figure S1: NMR analyses of Omnivin WG (***Vv***): (A) ^{31}P NMR; (B) ^{1}H-^{13}C HSQC.

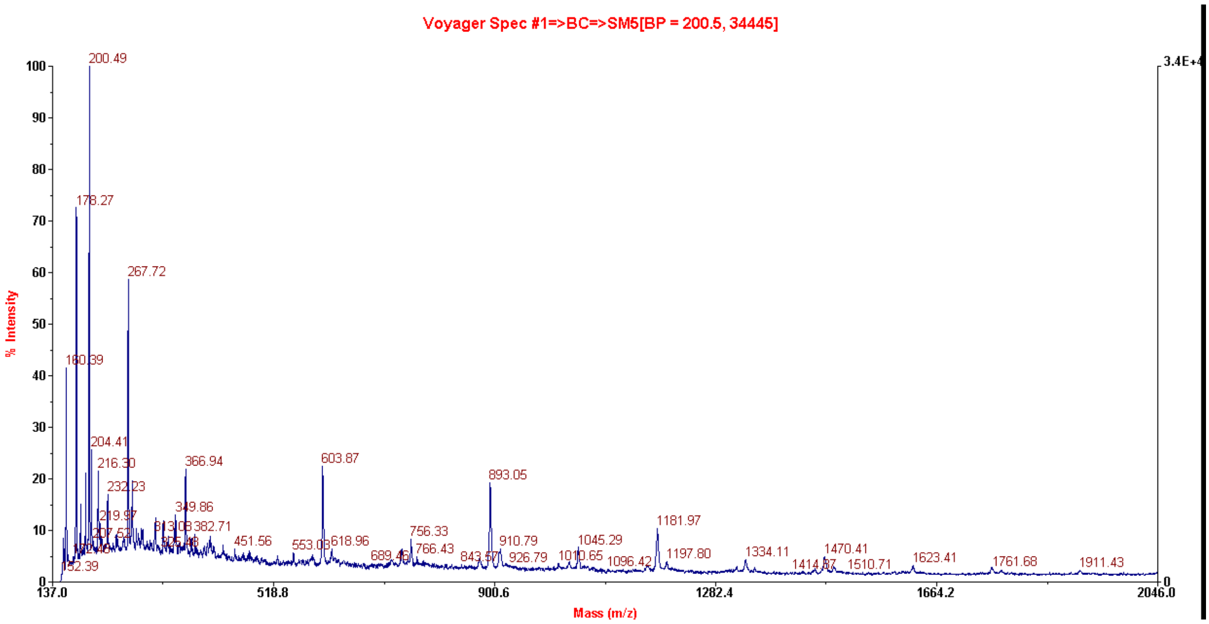

Figure S2: MALDI-ToF analysis of Omnivin WG (***Vv***).

(A)

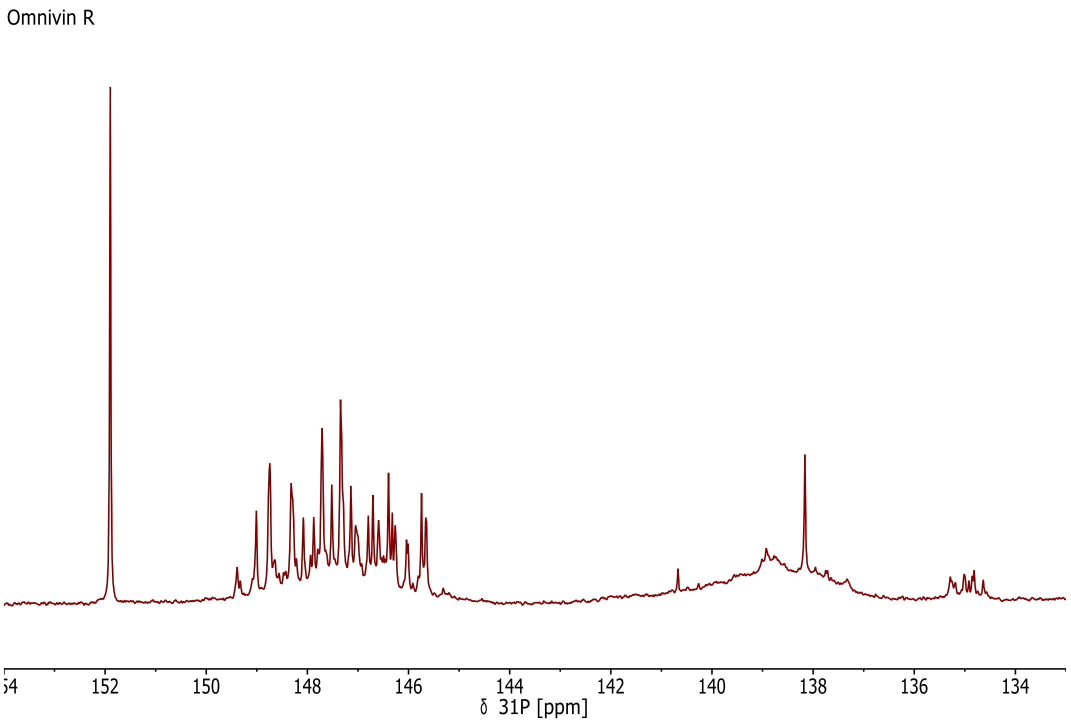

(B)

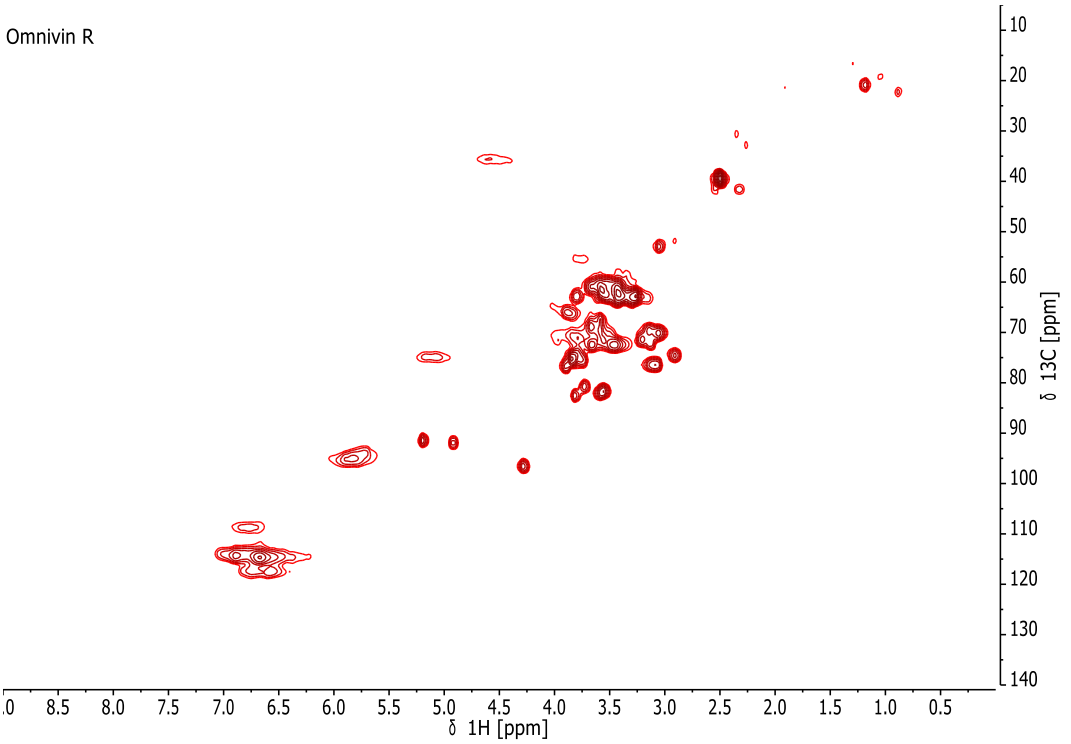

Figure S3: NMR analyses of Omnivin R (***Vv-R***): (A) ^{31}P NMR; (B) ^{1}H-^{13}C HSQC.

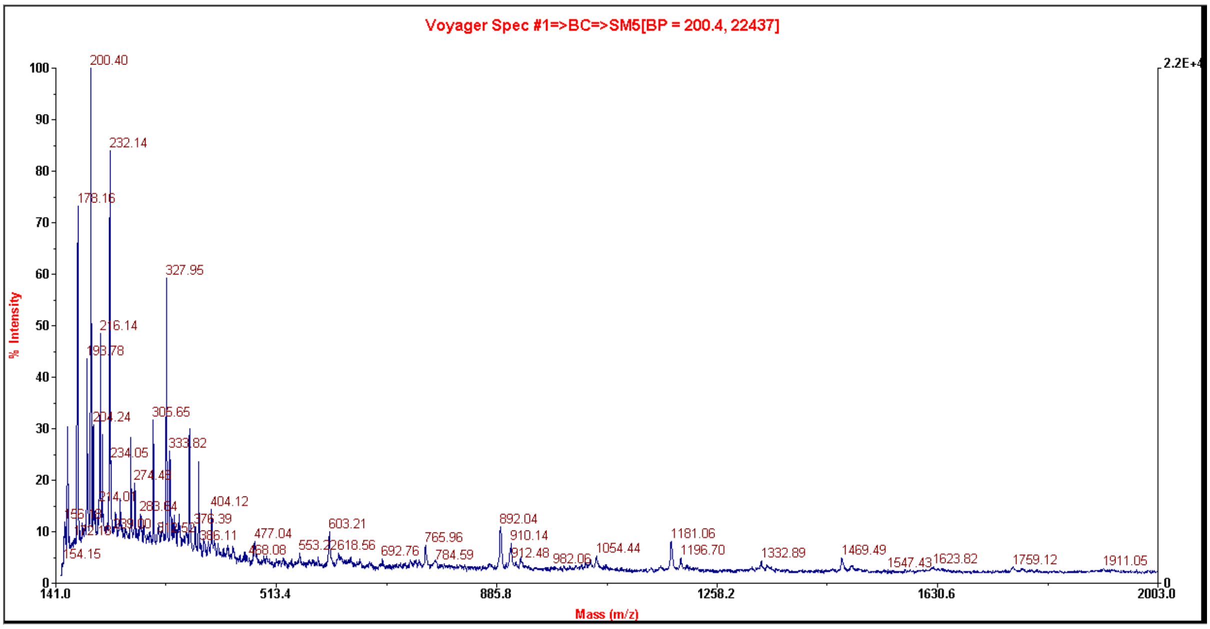

Figure S4: MALDI-ToF analysis of Omnivin R (***Vv*-R**).

(A)

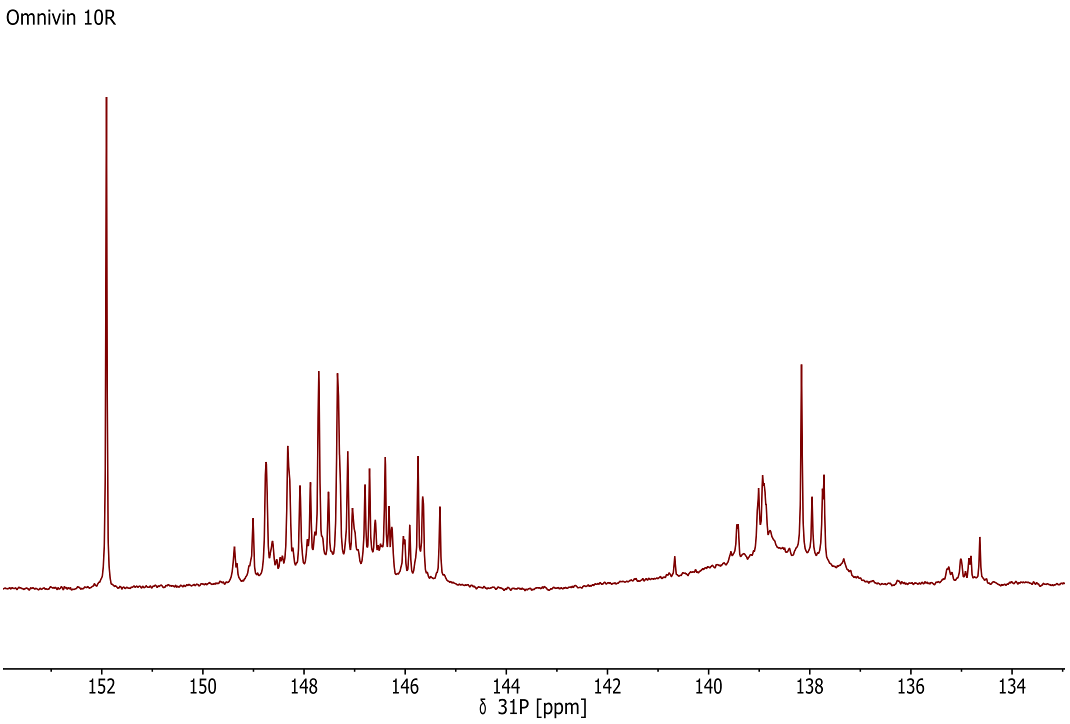

(B)

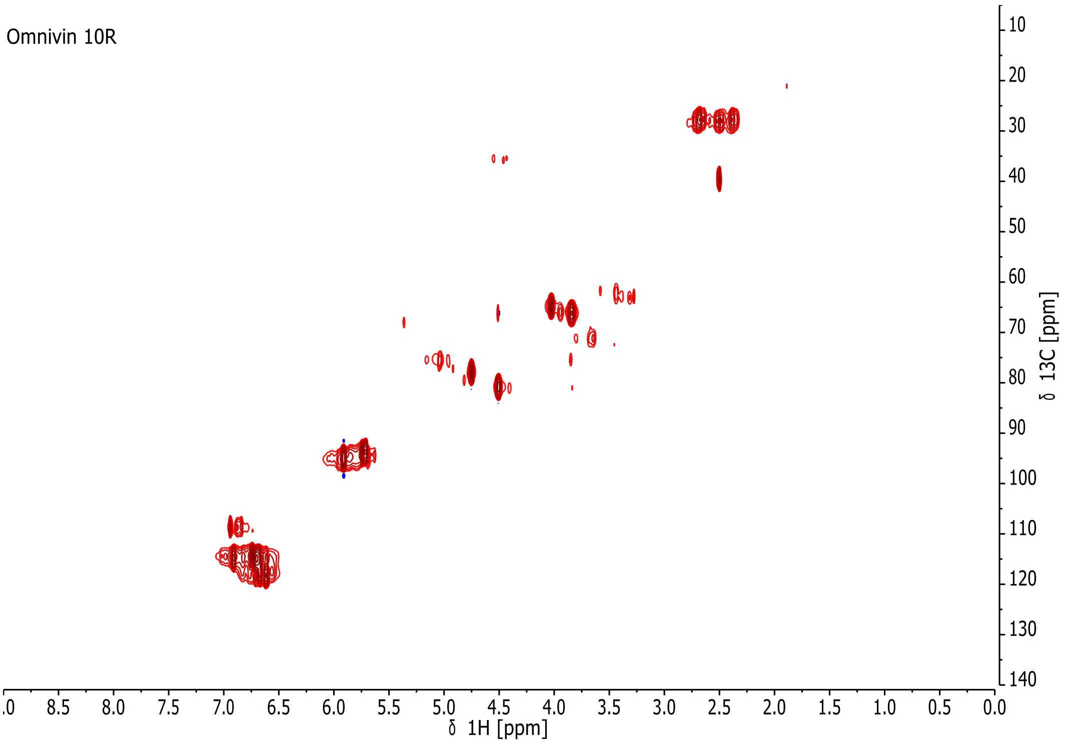

Figure S5: NMR analyses of Omnivin 10R (***Vv*-10**): (A) ^{31}P NMR; (B) ^{1}H-^{13}C HSQC.

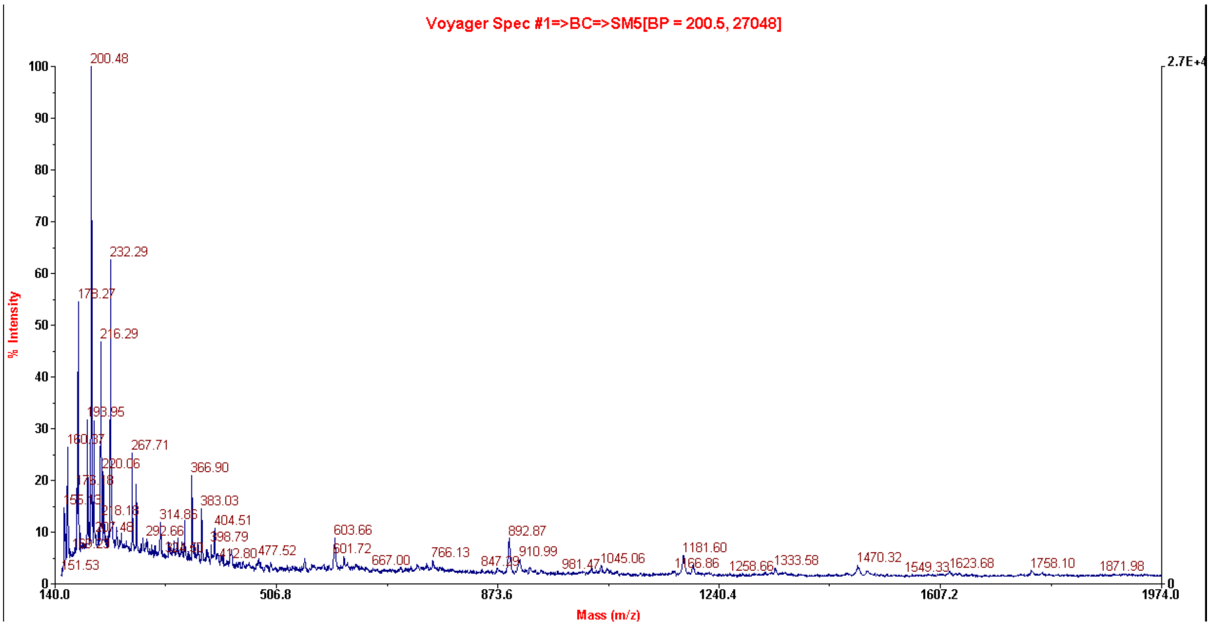

Figure S6: MALDI-ToF analysis of Omnivin 10R (***Vv*-10**).

(A)

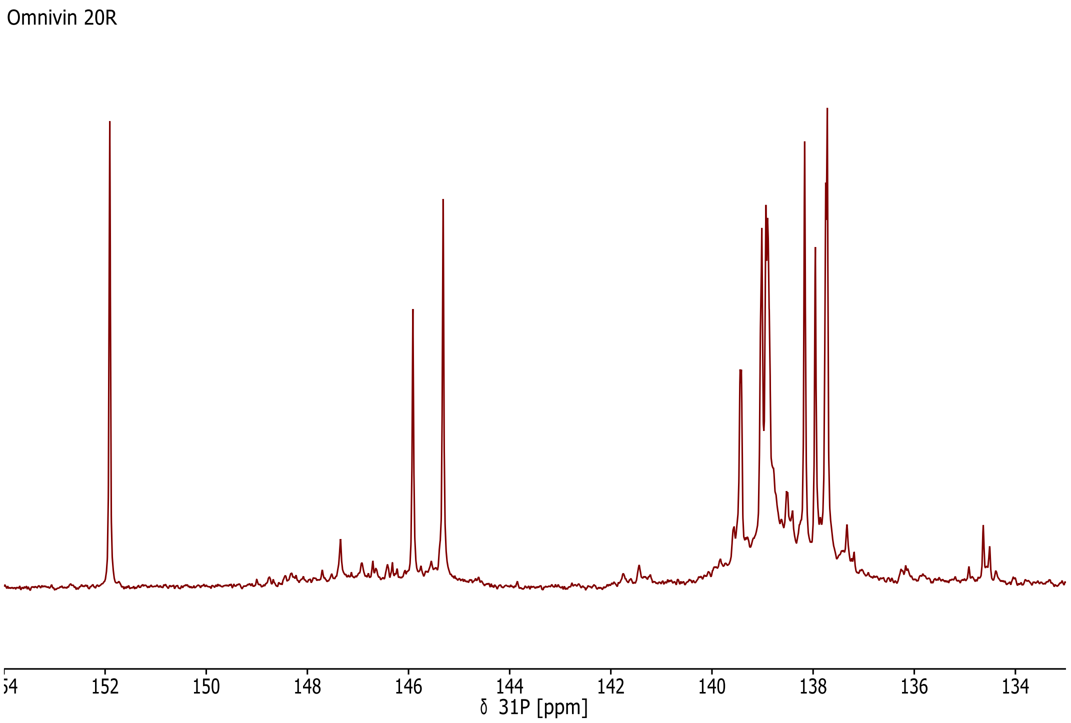

(B)

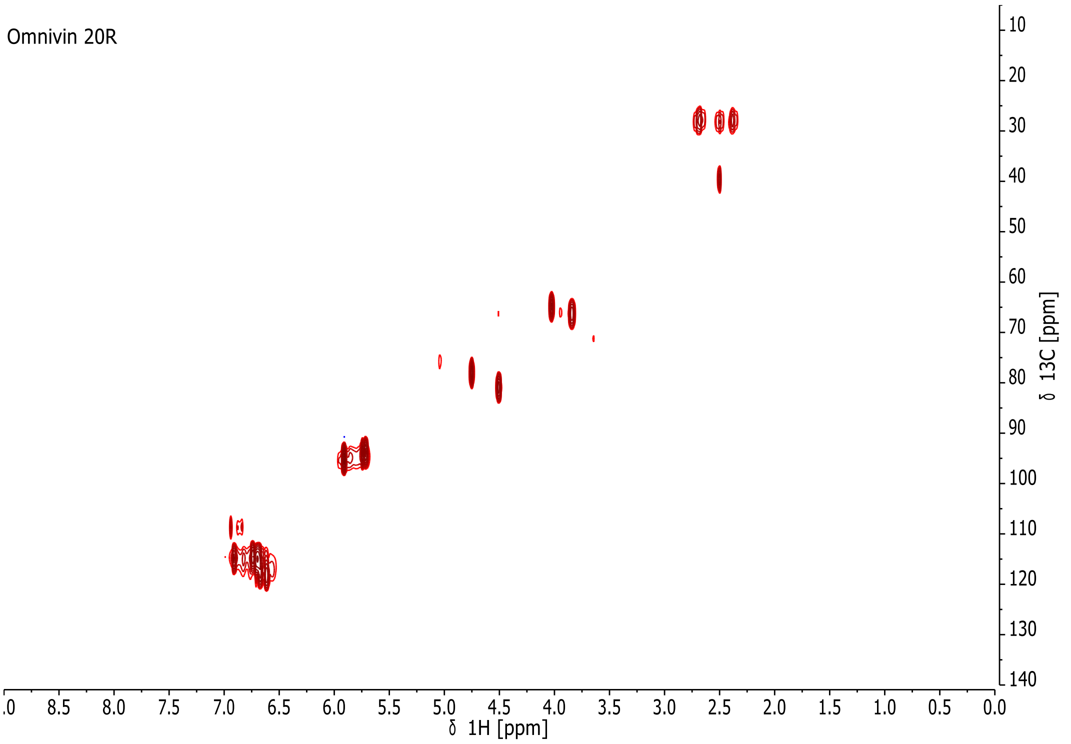

Figure S7: NMR analyses of Omnivin R20 (***Vv*-20**): (A) ^{31}P NMR; (B) ^{1}H-^{13}C HSQC.

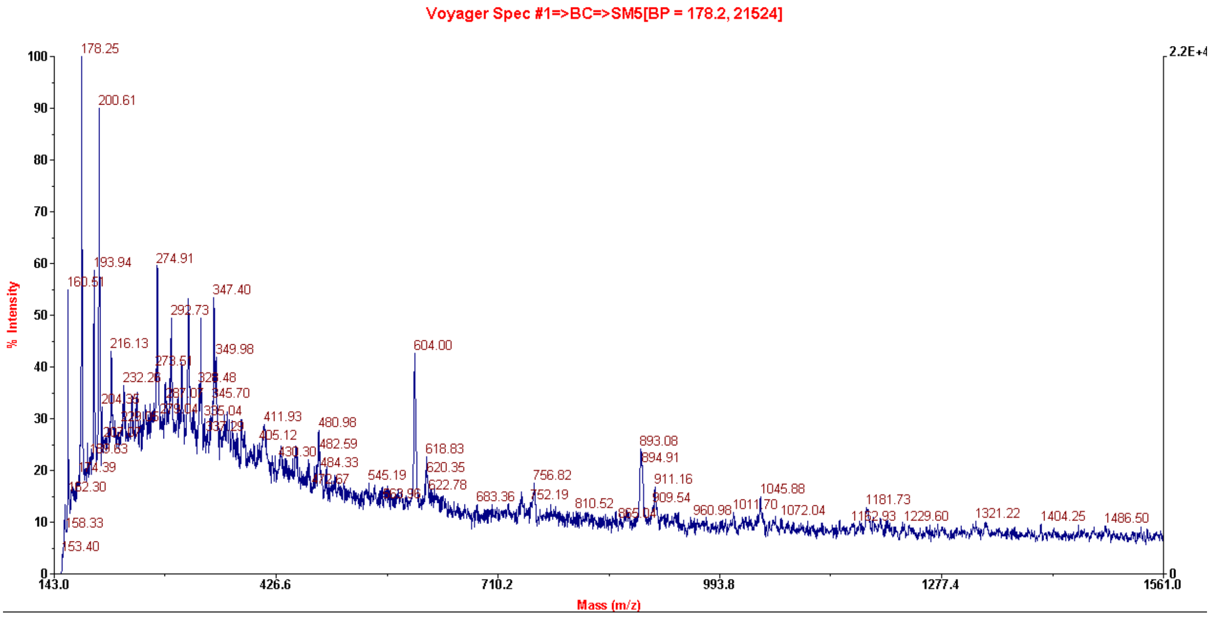

Figure S8: MALDI-ToF analysis of Omnivin 20R (***Vv*-20**).

(A)

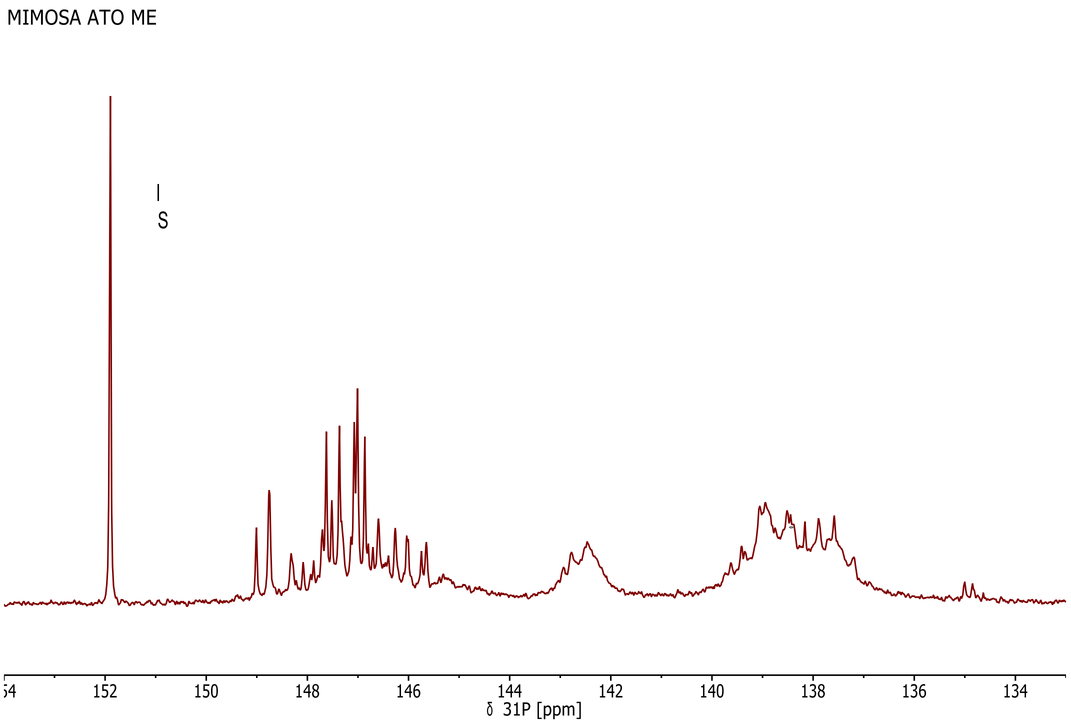

(B)

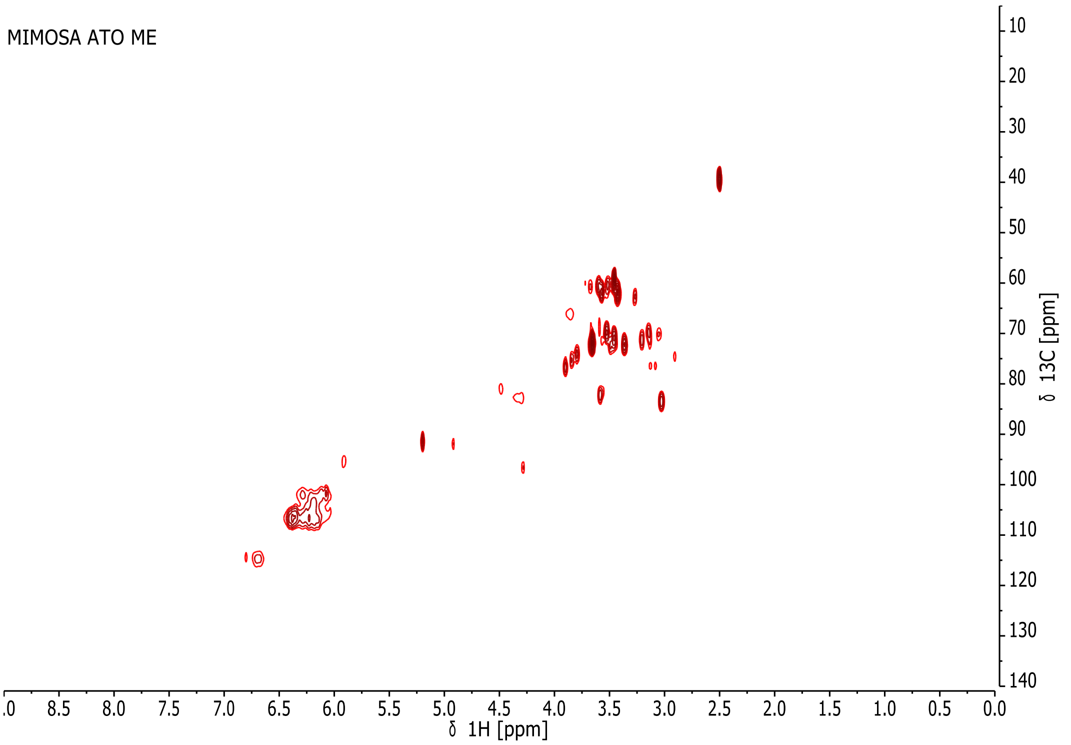

Figure S9: NMR analyses of MIMOSA ATO ME (***Am***): (A) ^{31}P NMR; (B) ^{1}H-^{13}C HSQC.

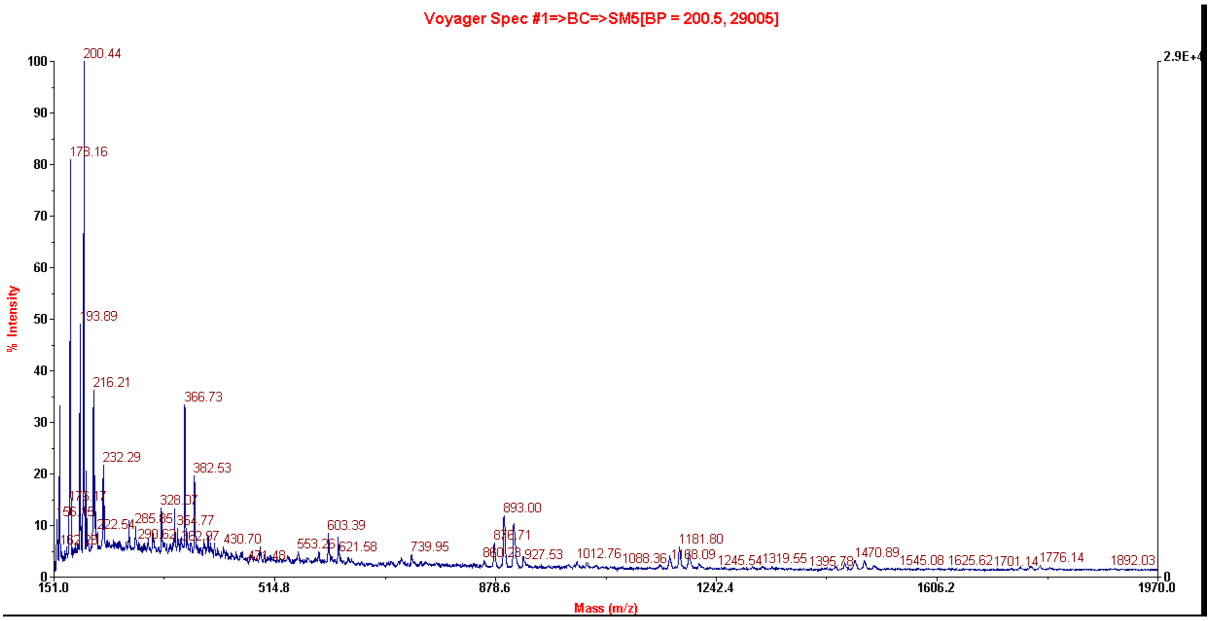

Figure S10: MALDI-ToF analysis of MIMOSA ATO ME (***Am***).

(A)

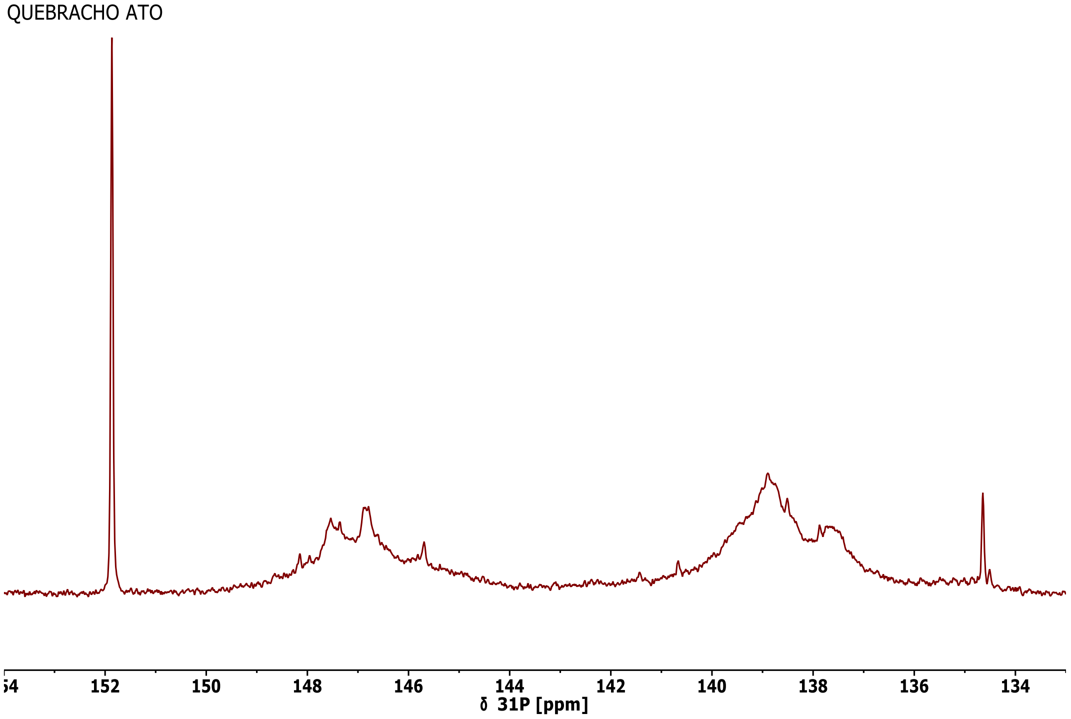

(B)

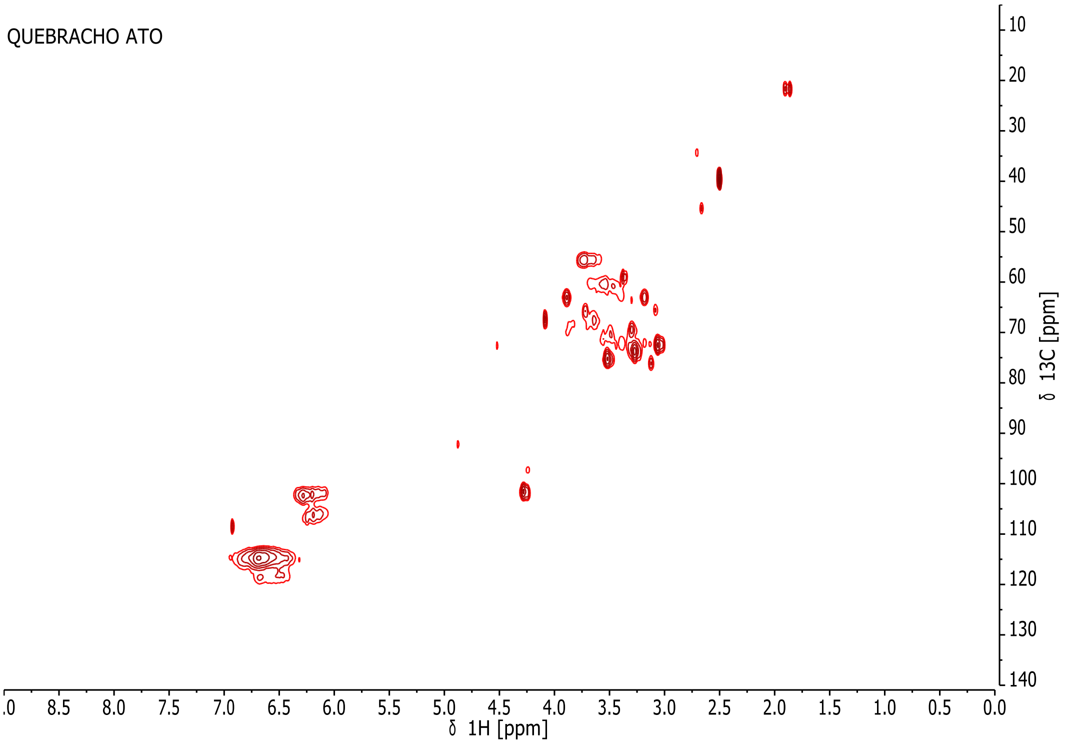

Figure S11: NMR analyses of QUEBRACHO ATO (***Sb***): (A) ^{31}P NMR; (B) ^{1}H-^{13}C HSQC.

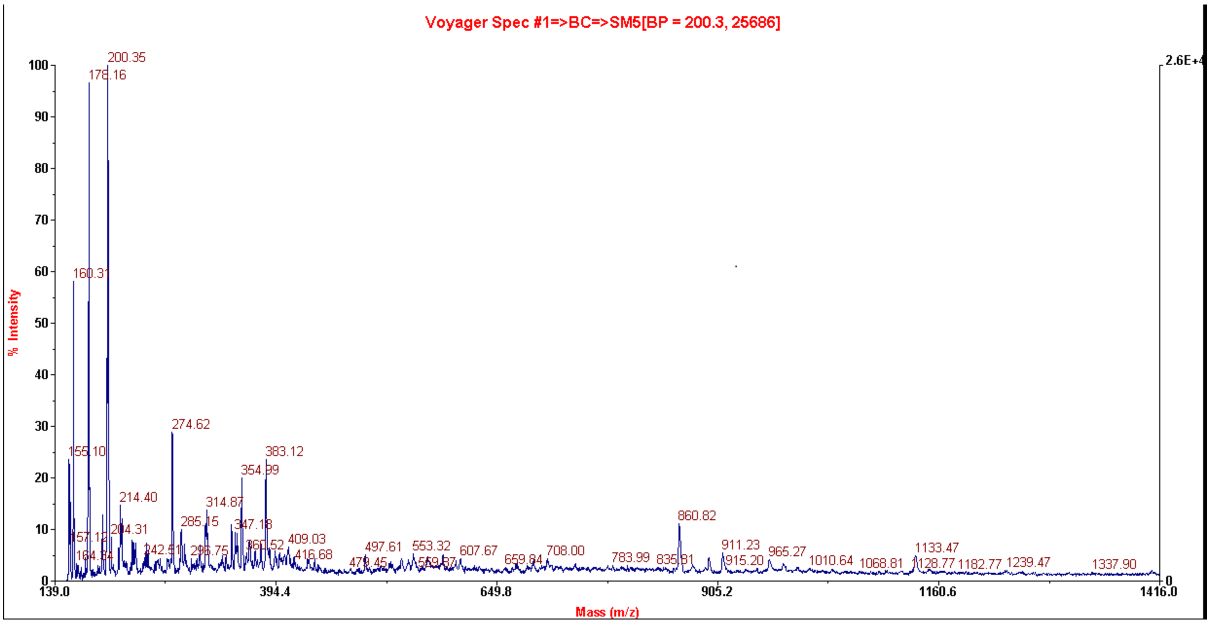

Figure S12: MALDI-ToF analyses of QUEBRACHO ATO (***Sb***).

Table S1: Results of qualitative ^{1}H-^{13}C HSQC analyses of commercialised condensed tannins according to literature reports;[1–5] atom numbering follows standard literature conventions.

Assignment	Cross-peak (δ[ppm] ^{1}H / δ[ppm] ^{13}C)					
	Vv	***Vv-R***	***Vv-10***	***Vv-20***	***Am***	***Sb***
C4-H, free, (epi)(gallo) catechin-*O*-3-gallate	2.65/27.53	3.04/27.26		3.03/25.22<br>2.29/25.72		
C4-H, β, free,(epi)catechin		2.69/27.87	2.67/27.74	2.68/27.71	2.66/28.06	2.73/28.74
C4-H, α, free, (epi)catechin	2.47/27.80<br>2.35/27.63	2.49/27.29<br>2.36/27.45	2.49/27.74<br>2.36/28.14	2.49/27.82<br>2.38/27.88	2.47/28.00<br>2.37/27.76	2.50/28.77
C4-H, α linked C4 or C8	4.52/35.33	4.62/35.66	4.59/35.51	4.55/35.26<br>4.46/35.37	4.78/35.50<br>4.73/35.16	4.69/35.24
C4-H, β linked C4 or C8	4.40/35.44<br>4.43/35.58	4.41/35.92	4.43/35.79	4.43/35.18<br>4.34735.92	4.68/35.35<br>4.60/35.64<br>4.56/35.97	4.64/35.30
C3-H, epicatechin monomer	4.02/64.49	4.02/64.64	4.02/64.52	4.02/64.72		
C3-H, epigallocatechin monomer	3.92/65.58			3.94/65.68		3.89/63.13<br>3.72/65.82
C3-H, catechin monomer	3.83/65.83	3.88/66.02	3.85/65.82	3.84/66.08	3.85/66.39	
C3-H, linked, terminal in dimers	3.56/68.82<br>3.67/69.06	3.67/68.83<br>3.59/69.69	3.66/69.58	3.65/70.83	3.59/68.83	3.64/67.70
C3-H, linked, terminal in oligomers			3.58/69.33		3.53/70.07	3.49/70.38
C3-H, linked, internal in oligomers		3.56/71.57<br>3.45/72.35	3.45/72.35		3.46/71.11<br>3.49/72.63<br>3.36/72.45	3.38/72.17<br>3.27/73.80
C2-H, epicatechin-*O*-3-gallate	5.20/74.85<br>5.03/74.86	5.17/74.85<br>5.08/74.87	5.14/74.77<br>5.07/74.93	5.04/75.17		
C2-H, (epi)gallocatechin-*O*-3-gallate	5.00/75.06<br>4.92/75.36				5.14/80.42<br>5.12/80.55<br>4.97/79.87	
C2-H, *cis*, epicatechin	4.75/77.80	4.75/77.80	4.74/77.55	4.75/77.76		
C2-H, *cis*, epigallocatechin	4.78/79.49				4.75/79.13	
C2-H, *cis*, epicatechin dimer				4.82/78.88		
C2-H, *cis*, epicatechin, oligomer		4.82/79.89				
C2-H, *trans*, catechin	4.49/80.53	4.48/80.77	4.49/80.62	4.50/80.72		4.62/80.00
C2-H, *trans*, catechin dimer	4.49/80.53			4.41/81.52		4.42/82.44
C2-H, *trans*, gallocatechin	4.37/80.85				4.59/80.44<br>4.56/81.05<br>4.49/81.28<br>4.30/83.10	
C2-H, *trans*, catechin oligomer		4.29/81.89				
C8-H, A ring, phloroglucinol	5.68/93.49<br>5.70/93.72<br>5.81/94.65	5.84/95.04	5.71/93.82	5.72/93.54<br>5.74/93.77	5.71/93.89<br>5.79/95.50	
C6-H, A ring, phloroglucinol	5.88/94.69		5.90/94.80	5.91/94.77	5.92/95.66<br>6.09/94.73	5.93/94.87<br>5.92/95.97<br>6.00/94.73
C8-H, A ring, resorcinol					6.29/102.25<br>6.12/102.24<br>6.07/102.05	6.28/102.53<br>6.20/102.34
C2'-H, C6'-H, B ring, pyrogallol;<br>A ring C6-H resorcinol					6.57/106.26<br>6.38/107.02<br>6.23/107.00<br>5.88/105.37	6.35/107.22<br>6.25/106.85<br>6.19/106.34<br>6.13/106.26

Assignment	Cross-peak (δ[ppm] ^{1}H / δ[ppm] ^{13}C)					
	Vv	***Vv-R***	***Vv-10***	***Vv-20***	***Am***	***Sb***
C2"-H, C6"-H, D ring	6.81/108.33<br>6.91/108.33	6.83/108.73<br>6.78/108.77	6.84/108.49,<br>6.79/108.55	6.94/108.41<br>6.87/108.48<br>6.84/108.41		
C2'-H, C6'-H, B ring, pyrogallol	6.88/110.87	6.80/110.85				
C2'-H, C6'-H, B ring, phloroglucinol						
C2'-H, B ring, catechol	6.87/114.39	6.89/114.38	6.90/114.39	6.91/114.58	6.80/114.71	
C5'-H, B ring, catechol	6.66/114.58	6.68/114.70	6.69/114.75	6.74/114.26<br>6.70/114.72	6.70/115.08<br>6.56/114.89<br>6.42/115.42	6.69/114.95
C2"-H, C6"-H, D ring, oligomer internal	7.07/108.70	7.55/114.39<br>7.47/114.19	7.33/114.35	7.46/114.35	6.97/108.55	6.93/108.67
C6'-H, B ring, catechol	6.58/117.78	6.74/117.29	6.61/119.27	6.62/117.98	6.71/119.35	6.69/118.69
C5-H, A ring, resorcinol					6.43/128.73	6.44/130.11

Table S2: MALDI-ToF analysis of commercialised condensed tannins. For letter codes of identified monomeric building blocks refer to Figure 2 in the main article.

Tannin	Observed mass peak [Da]	Calculated mass [Da]	Assignment
Vv [a]	314.8	313.3	A+Na
	328.3	329.3	B+Na
	603.9	601.6	AA+Na
	619.0	617.6	AB+Na
	756.3	754.7	AAG+Na
Vv-R [a]	328.0	329.3	B+Na
	603.2	601.6	AA+Na
	618.6	617.6	AB+Na
	892.0	889.9	AAA+Na
	1044.9	1042.0	AAAG+Na
Vv-10 [a]	314.9	313.3	A+Na
	603.7	601.6	AA+Na
	766.1	769.7	ABG+Na
	892.9	889.9	AAA+Na
	1045.1	1042.0	AAAG+Na
Vv-20 [a]	314.4	313.3	A+Na
	328.2	329.3	B+Na
	466.6	465.4	AG+Na
	480.3	481.4	BG+Na
	603.6	601.6	AA+Na
	618.7	617.6	AB+Na
Am	274.6	275.3	C+H
	313.0	313.3	D+Na
	328.1	329.3	B+Na
	587.3	585.6	CD+Na
	603.4	601.6	DD+Na
	619.5	617.6	DB+Na
	723.7	721.7	CCG+Na
	740.0	737.7	CDG+Na
	860.2	857.9	CCD+Na
	876.7	873.9	CDD+Na
	924.3	921.9	DBB+Na
	1012.8	1010.0	CCDG+Na
	1149.3	1146.2	CCDD+Na
	1165.6	1162.2	CDDD+Na
	1181.8	1178.2	DDDD+Na
	1197.7	1194.2	DDDB+Na
	1213.2	1210.2	DDBB+Na
Sb [a]	274.6	275.3	C+H
	314.1	313.3	A+Na
	547.5	547.6	CC+H
	563.6	563.6	AC+H
	569.7	569.6	CC+Na
	587.4	585.6	AC+Na
	602.5	601.6	AA+Na
	721.9	721.7	CCG+Na
	740.7	738.7	ACG+Na
	753.0	754.7	AAG+Na
	890.4	889.9	AAA+Na
	1133.5	1130.2	ACCC+Na

[a] Free gallic acid detected in sample.

Analysis data for commercialised hydrolysable tannins

(A)

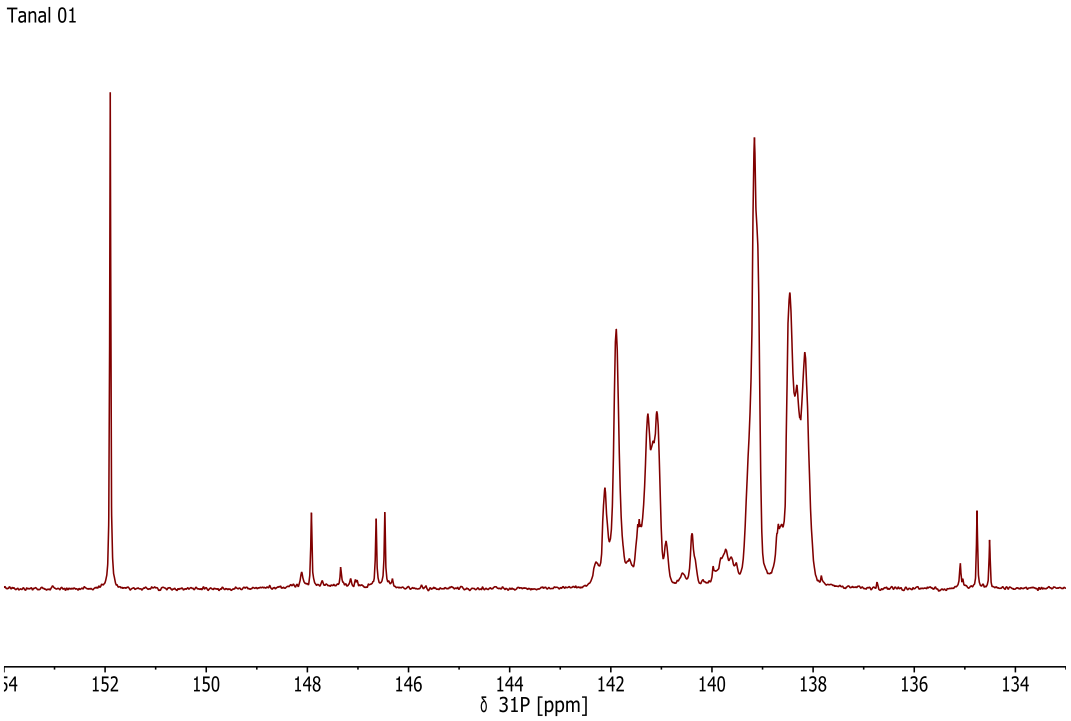

(B)

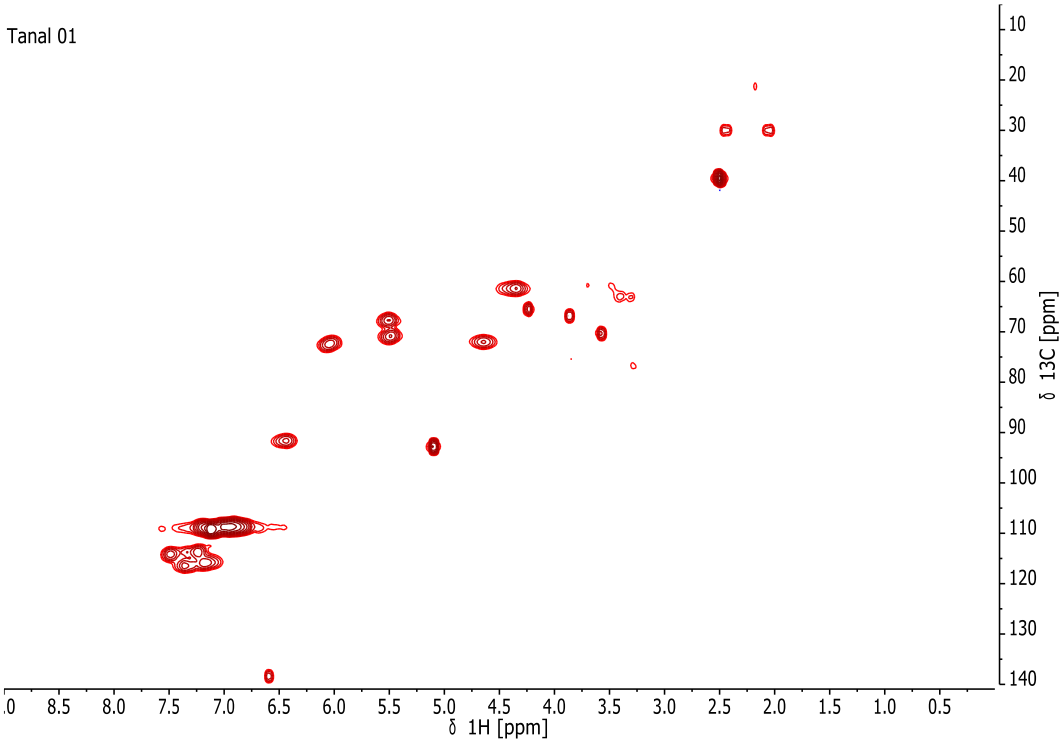

Figure S13: NMR analyses of Tanal 01 (**Ta-01**): (A) ^{31}P NMR; (B) ^{1}H-^{13}C HSQC.

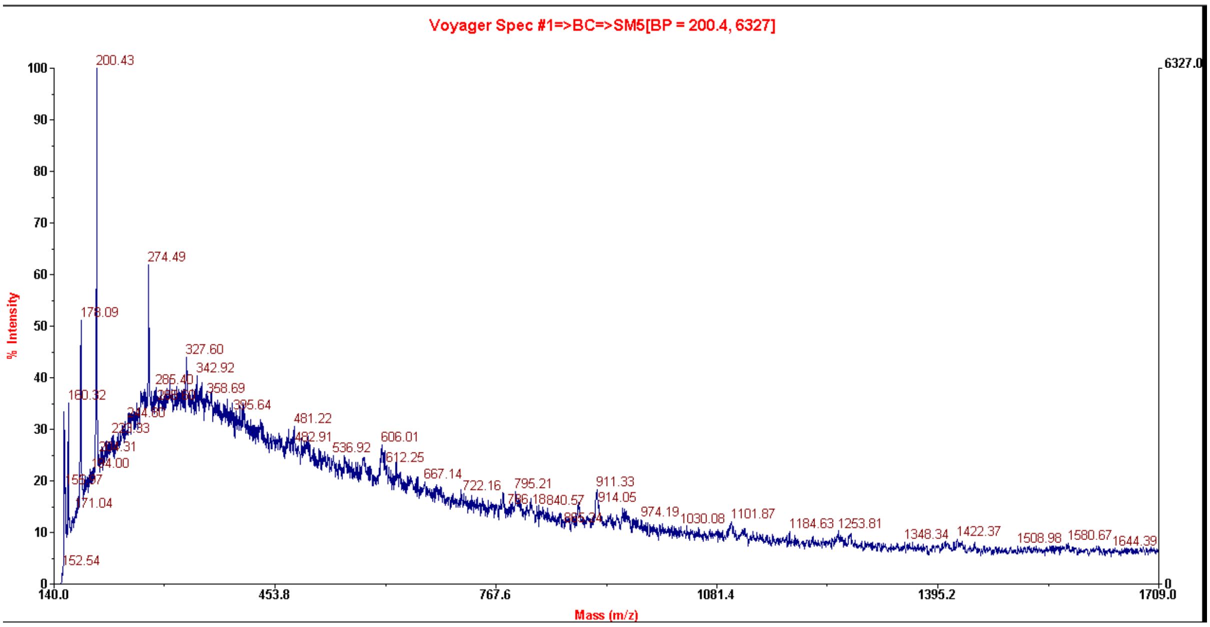

Figure S14: MALDI-ToF analyses of Tanal-01 (**Ta-01**).

(A)

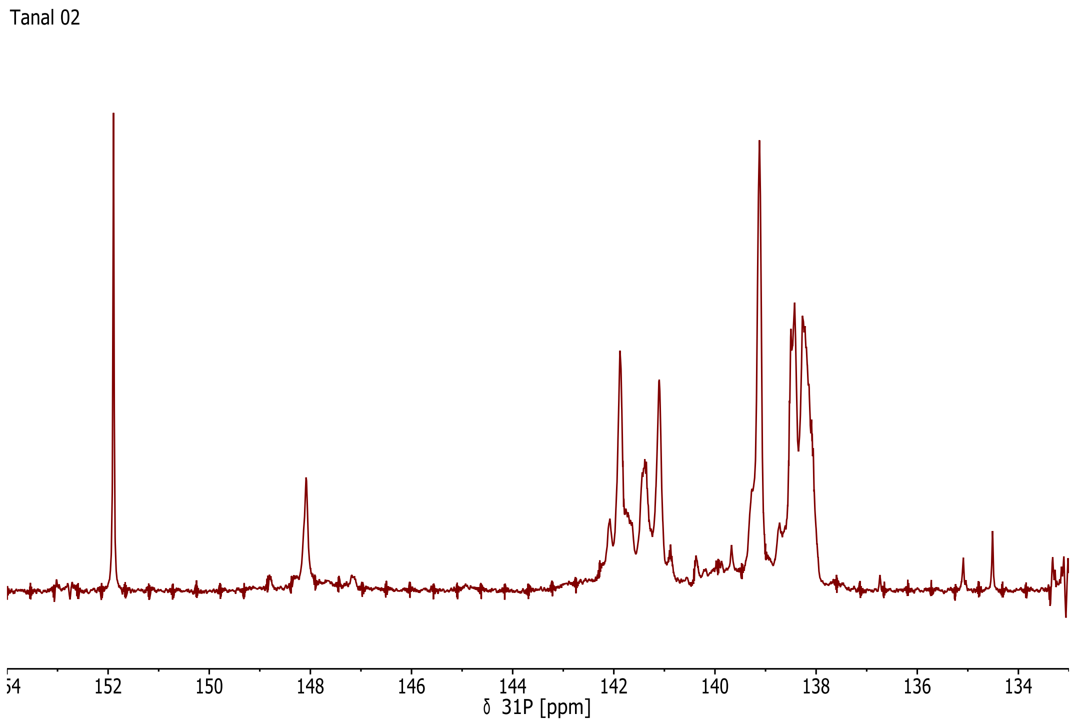

(B)

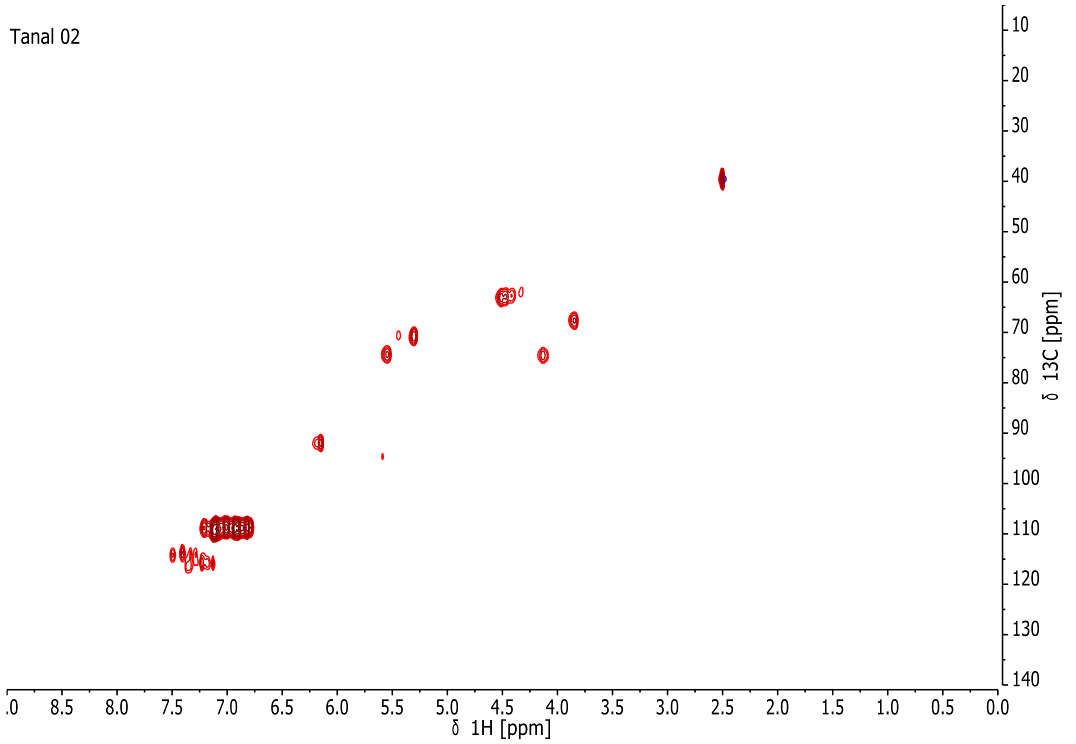

Figure S15: NMR analyses of Tanal 02 (**Ta-02**): (A) ^{31}P NMR; (B) ^{1}H-^{13}C HSQC.

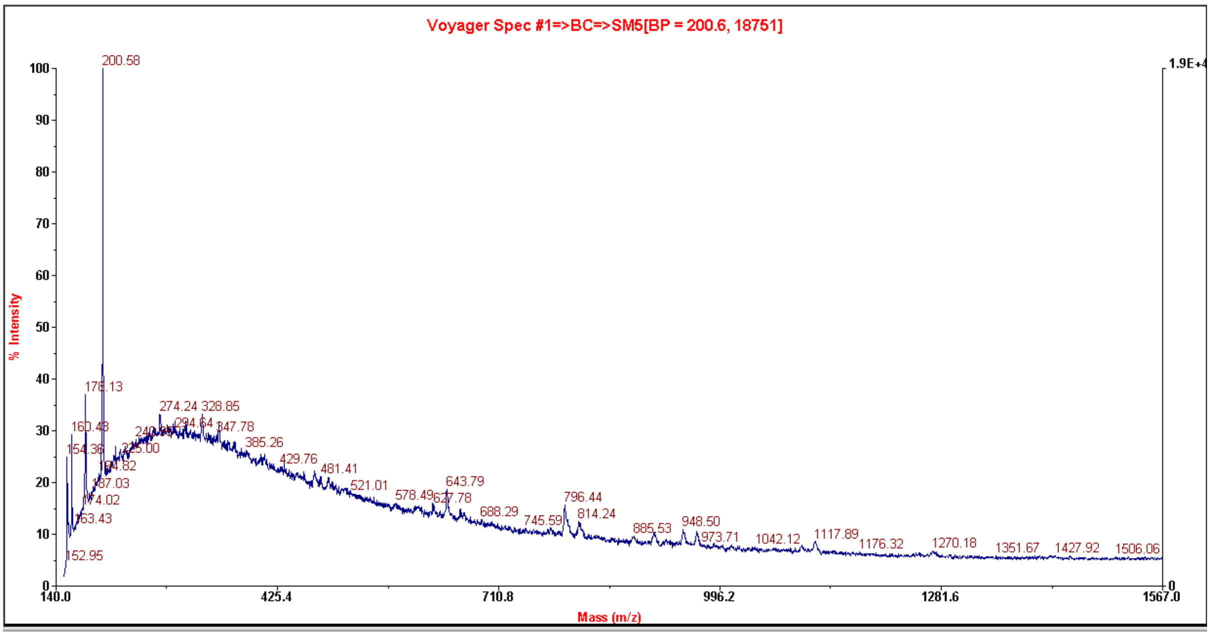

Figure S16: MALDI-ToF analyses of Tanal-01 (**Ta-02**).

(A)

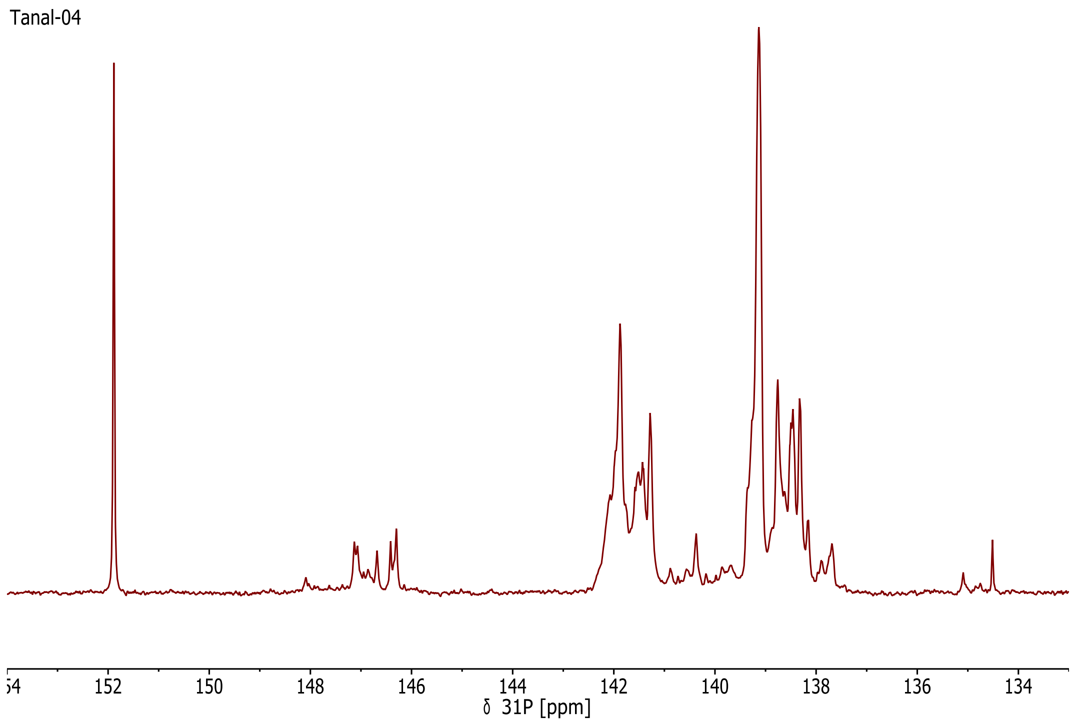

(B)

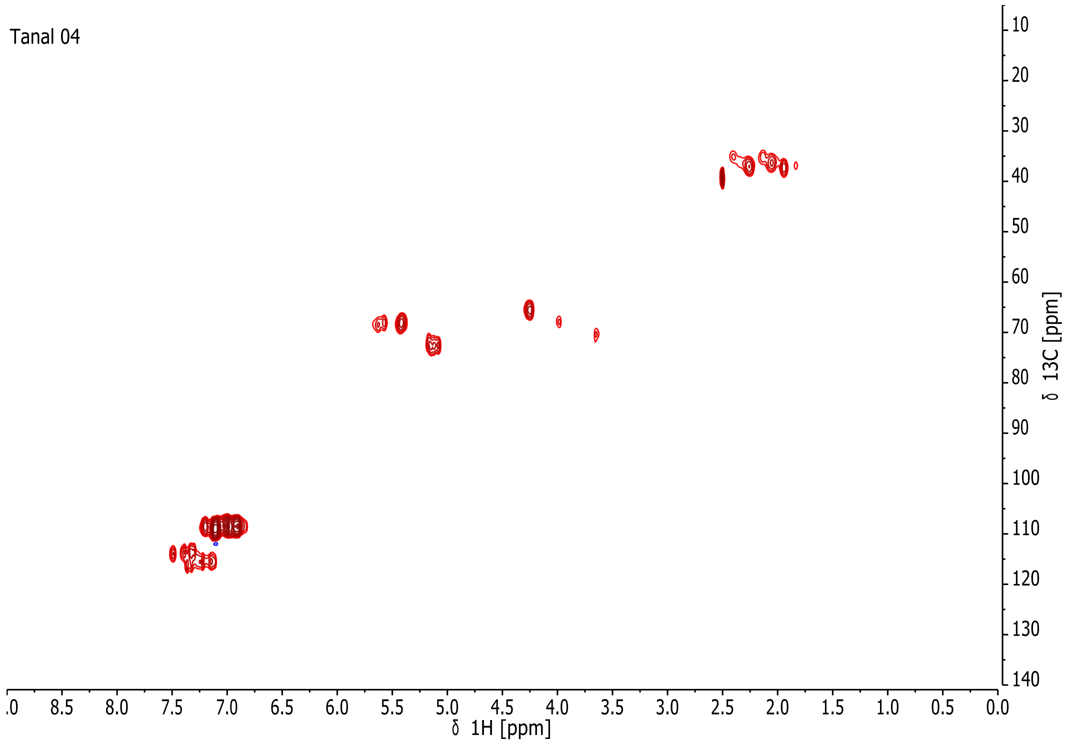

Figure S17: NMR analyses of Tanal 04 (**Ta-04**): (A) ^{31}P NMR; (B) ^{1}H-^{13}C HSQC.

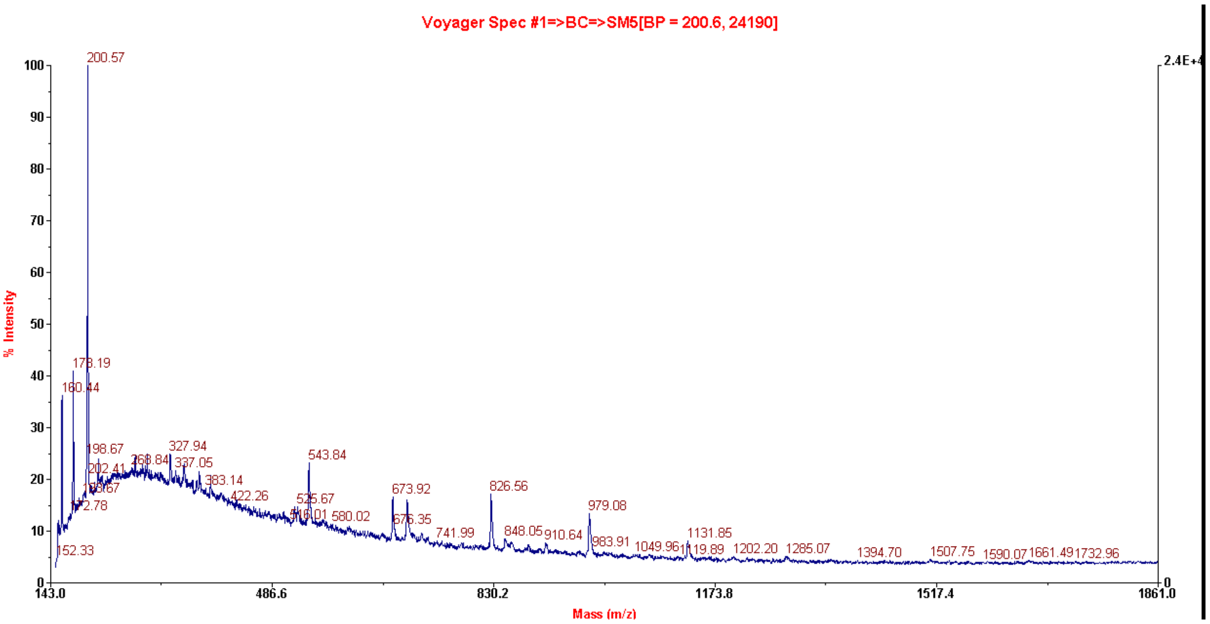

Figure S18: MALDI-ToF analyses of Tanal-01 (**Ta-04**).

(A)

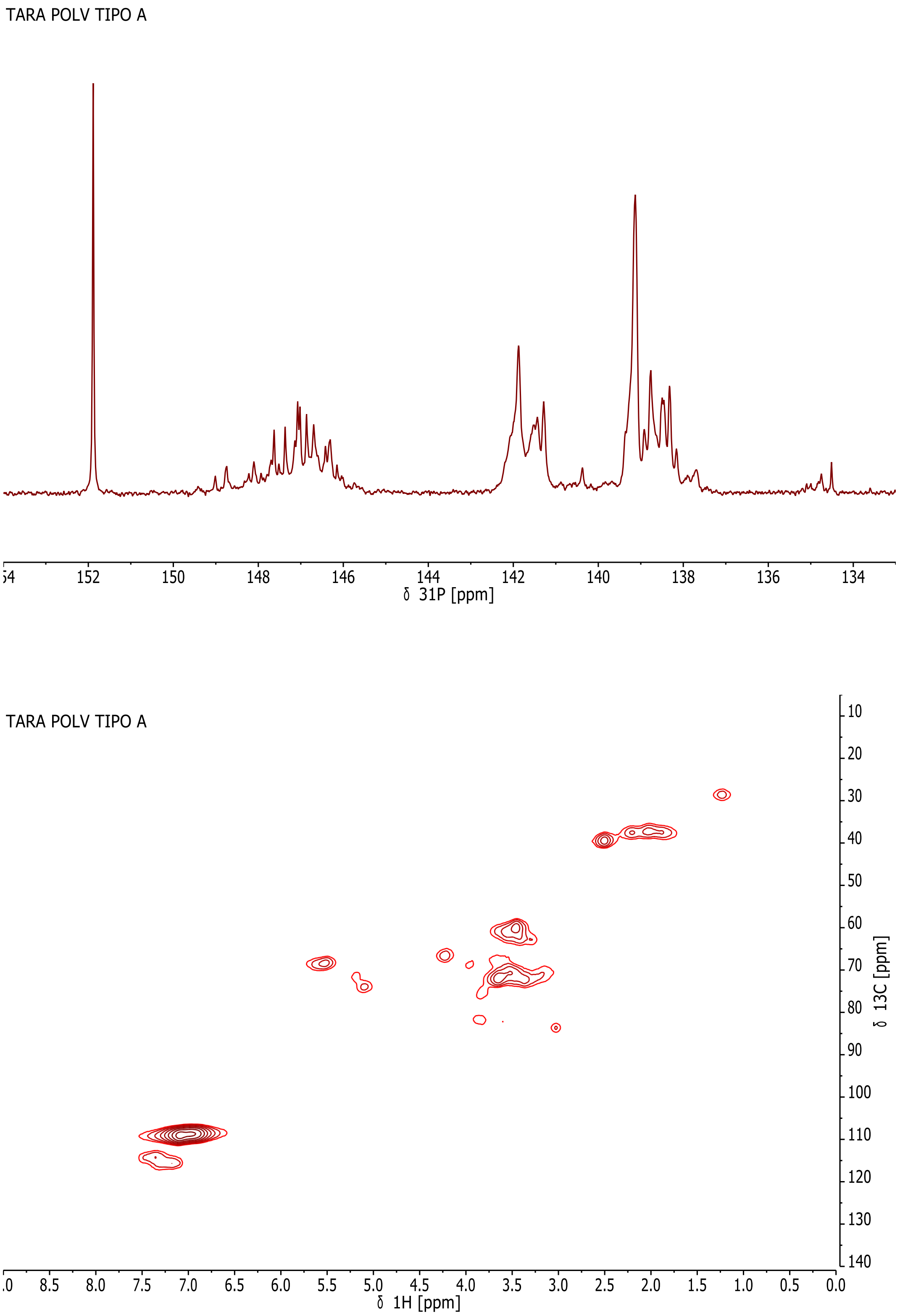

(B)

Figure S19: NMR analyses of TARA POLV TIPO A (***Ct***): (A) ^{31}P NMR; (B) ^{1}H-^{13}C HSQC.

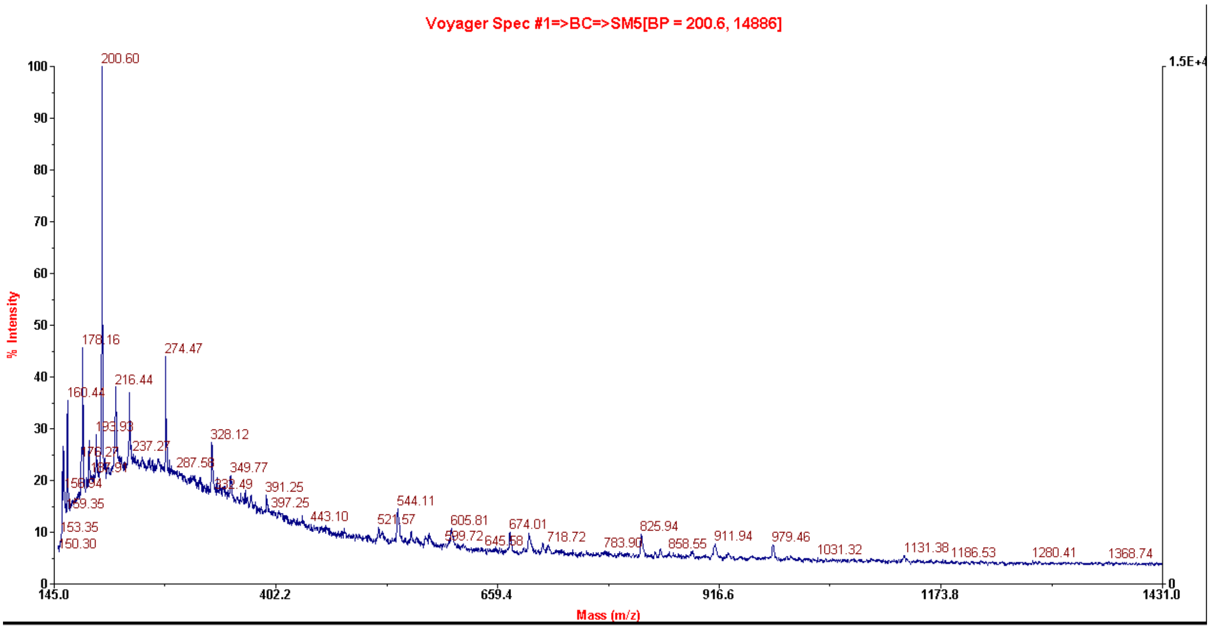

Figure S20: MALDI-ToF analyses of TARA POLV TIPO A (***Ct***).

(A)

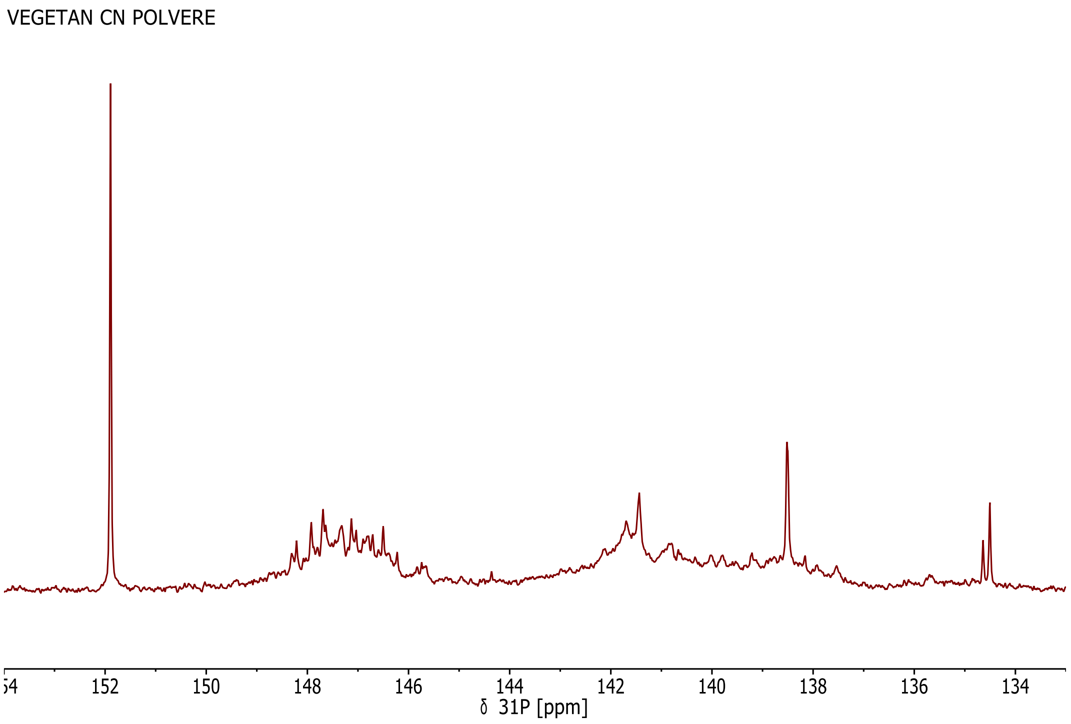

(B)

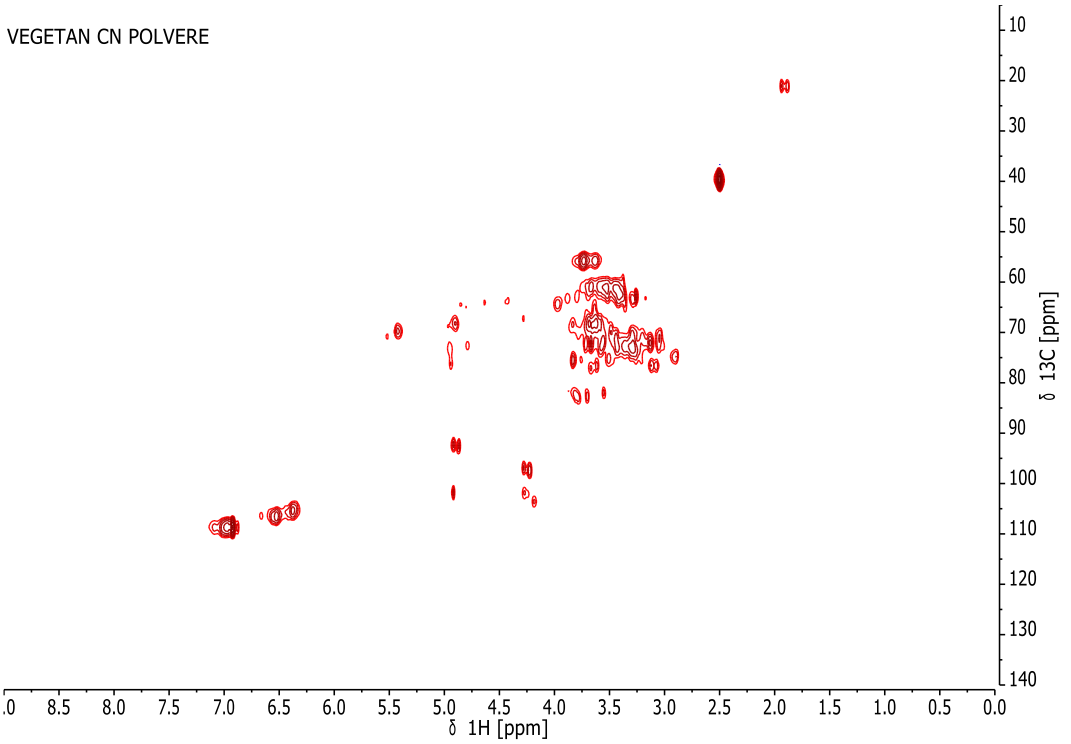

Figure S21: NMR analyses of VEGETAN CN POLVERE (***Cs***): (A) ^{31}P NMR; (B) ^{1}H-^{13}C HSQC.

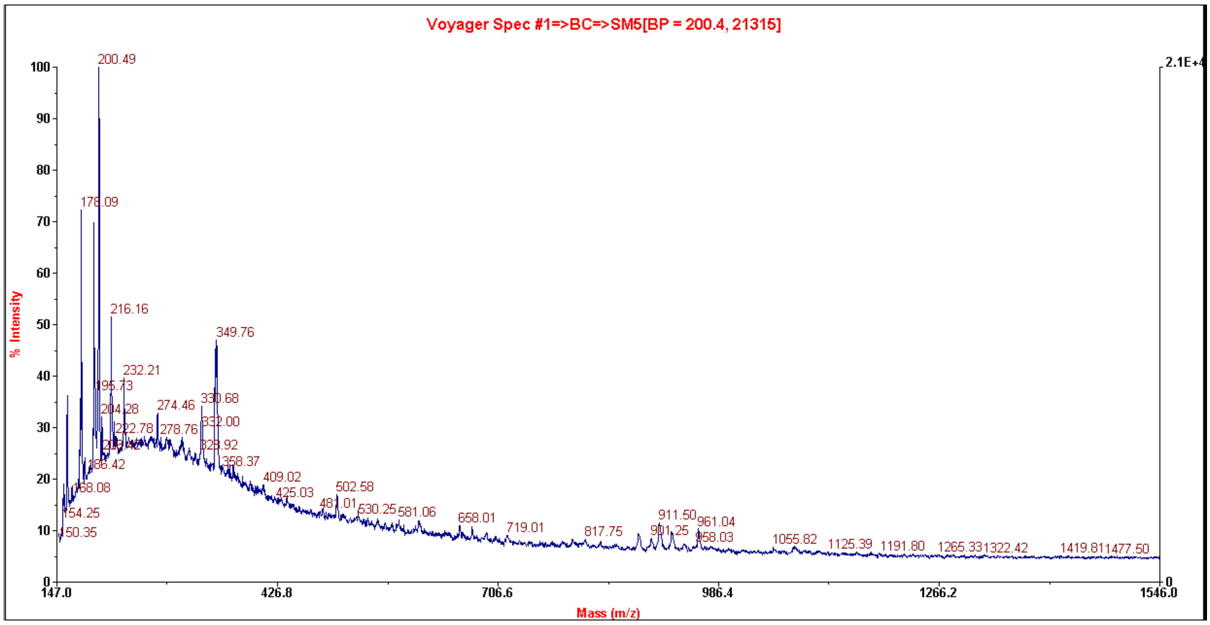

Figure S22: MALDI-ToF analyses of VEGETAN CN POLVERE (***Cs***).

Table S3: Results of qualitative ^{1}H-^{13}C HSQC analyses of commercialised hydrolysable tannins according to literature reports;[1–5] atom numbering according to standard literature conventions for carbohydrates and gallic acid moieties.

Assignment	Cross-peak (δ[ppm] ^{1}H / δ[ppm] ^{13}C)				
	Ta-01	***Ta-02***	***Ta-04***	***Ct***	***Cs***
quinic acid, C2-H			2.13/35.47<br>2.05/36.62	2.03/37.37	
quinic acid, C6-H			2.26/37.34	2.20/37.65	
glucopyranose, C6-Hα		4.54/63.11<br>4.50/62.95			6.85/64.43
glucopyranose, C6-Hβ		4.45/62.77<br>4.36/62.04			
glucopyranose, C6-H	4.35/61.25	4.54/63.11<br>4.50/62.95			
glucopyranose, C4-H	5.50/67.84	5.50/67.84			5.42/69.69
quinic acid, C3-H			5.57/68.41<br>5.63/68.82	5.51/68.53	
quinic acid, C5-H			5.42/68.42<br>5.29/71.33	5.18/71.66	
quinic acid, C4-H			5.08/72.09<br>5.15/72.14<br>5.12/72.98<br>5.15/73.12	5.08/74.11	
glucopyranose, C2-H	5.49/70.17	5.47/70.63			4.94/76.18
glucopyranose, C5-H	4.64/71.87	4.66/72.11			4.90/68.16
glucopyranose, C3-H	6.04/72.29	6.01/71.93			4.95/73.11
glucopyranose, C1-H	6.44/91.47	6.42/91.77			4.92/101.77
HHDP, C6-H,C6'-H					6.38/105.35
G-G-G					6.927108.63<br>6.97/108.59<br>6.99/104.57
hellinoyl, C2-H, C6-H, and hellinoyl, C6'-H	6.46/108.73	6.46/108.73	6.70/109.17		
terminal galloyl	6.95/108.59	7.04/108.75<br>6.93/108.99<br>6.90/108.90<br>6.85/108.82	6.92/108.87<br>6.99/108.81<br>6.92/108.89	6.97/108.81<br>7.08/109.16	
flavogallonic acid,C6"-H	7.17/109.03	7.24/108.87<br>7.14/109.13	7.20/108.93<br>7.10/109.31		
ellagic acid, C5H5,C5"-H"	7.56/108.96	7.40/108.94<br>7.56/109.40	7.39/109.33		7.44/109.50
flavogallonic acid,C5'-H	7.49/113.98	7.44/113.79,<br>7.53/114.28	7.49/114.34		
internal galloyl	7.24/113.59<br>7.17/115.29	7.16/115.94<br>7.26/115.79<br>7.32/114.15<br>7.21/115.84	7.30/114.05<br>7.39/114.01<br>7.33/114.14<br>7.14/115.87<br>7.20/115.95	7.34/114.33<br>7.17/115.68	
hellinoyl, C6"-H	7.36/116.29	7.38/116.48	7.36/116.58		

Table S4: MALDI-ToF analysis of commercialised hydrolysable tannins. Ln= gallotannin with n galloyl units; Qm = galloquinic acid with m galloyl units. For mentioned motifs see Figure 2 in the main article.

Tannin	Observed mass peak [Da]	Calculated mass [Da]	Assignment
**Ta-01**	172.7	171.1	gallic acid+H
	327.6	325.2	ellagic acid+Na
	356.5	355.3	L1+Na
	642.7	645.5	L7+2Na
	795.2	797.6	L9+2Na
	811.7	811.6	L44+Na
	1422.4	1420.0	L8+Na
**Ta-02**	175.4	171.1	gallic acid+H
	192.3	193.1	gallic acid+Na
	303.1	303.2	ellagic acid+H
	328.9	325.2	ellagic acid+Na
	492.5	493.3	flavogallonic acid +Na
	643.0	645.5	L7+2Na
	796.4	797.6	L9+2Na
	948.5	949.7	L11+2Na
	961.0	963.7	L5+Na
	1117.9	1115.8	L6+Na
	1270.2	1267.9	L7+Na
**Ta-04**	172.8	171.1	gallic acid+H
	327.9	325.2	ellagic acid+Na
	366.9	367.3	Q1+Na
	391.3	391.3	Q6+3Na
	471.3	471.3	flavogallonic acid+H
	494.3	493.3	flavogallonic acid +Na
	543.8	543.7	Q9+3Na
	673.9	671.5	Q3+Na
	696.4	695.8	Q12+3Na
	826.6	823.6	Q4+Na
	979.1	975.7	Q5+Na
	1131.9	1127.8	Q6+Na
Ct	174.0	171.1	gallic acid+H
	193.9	193.1	gallic acid+Na
	328.1	325.2	ellagic acid+Na
	366.7	367.3	Q1+Na
	521.6	519.4	Q2+Na
	544.1	543.4	Q9+3Na
	674.0	671.5	Q3+Na
	825.9	823.6	Q4+Na
	979.5	975.7	Q5+Na
	1131.4	1127.8	Q6+Na
Cs	193.9	193.1	gallic acid+Na
	303.1	303.2	ellagic acid+H
	502.6	505.4	HHDP+gluNa
	658.0	655.5	GGG+glu+Na
	961.0	957.6	castalgin / vescalgin

References

(1) Davis, A. L.; Cai, Y.; Davies, A. P.; Lewis, J. R. 1H and 13C NMR Assignments of Some Green Tea Polyphenols. *Magn. Reson. Chem.* **1996**, *34* (11), 887–890. https://doi.org/10.1002/(SICI)1097-458X(199611)34:11<887::AID-OMR995>3.0.CO;2-U.

(2) Konai, N.; Raidandi, D.; Pizzi, A.; Meva'a, L. Characterization of Ficus Sycomorus Tannin Using ATR-FT MIR, MALDI-TOF MS and 13C NMR Methods. *Eur. J. Wood Wood Prod.* **2017**, *75* (5), 807–815. https://doi.org/10.1007/s00107-017-1177-8.

(3) Thompson, D.; Pizzi, A. Simple 13C-NMR Methods for Quantitative Determinations of Polyflavonoid Tannin Characteristics. *J. Appl. Polym. Sci.* **1995**, *55* (1), 107–112. https://doi.org/10.1002/app.1995.070550111.

(4) de Souza, L. M.; Cipriani, T. R.; Iacomini, M.; Gorin, P. A. J.; Sassaki, G. L. HPLC/ESI-MS and NMR Analysis of Flavonoids and Tannins in Bioactive Extract from Leaves of Maytenus Ilicifolia. *J. Pharm. Biomed. Anal.* **2008**, *47* (1), 59–67. https://doi.org/10.1016/j.jpba.2007.12.008.

(5) Hoong, Y. B.; Pizzi, A.; Md. Tahir, P.; Pasch, H. Characterization of Acacia Mangium Polyflavonoid Tannins by MALDI-TOF Mass Spectrometry and CP-MAS 13C NMR. *Eur. Polym. J.* **2010**, *46* (6), 1268–1277. https://doi.org/10.1016/j.eurpolymj.2010.03.002.
